# VELCRO-IP RNA-seq reveals ribosome expansion segment function in translation genome-wide

**DOI:** 10.1016/j.celrep.2020.108629

**Published:** 2021-01-19

**Authors:** Kathrin Leppek, Gun Woo Byeon, Kotaro Fujii, Maria Barna

**Affiliations:** 1 Department of Developmental Biology, Stanford University, Stanford, CA 94305, USA; 2 Department of Genetics, Stanford University, Stanford, CA 94305, USA; 3 These authors contributed equally; 4 Lead contact

## Abstract

Roles for ribosomal RNA (rRNA) in gene regulation remain largely unexplored. With hundreds of rDNA units positioned across multiple loci, it is not possible to genetically modify rRNA in mammalian cells, hindering understanding of ribosome function. It remains elusive whether expansion segments (ESs), tentacle-like rRNA extensions that vary in sequence and size across eukaryotic evolution, may have functional roles in translation control. Here, we develop variable expansion segment-ligand chimeric ribosome immunoprecipitation RNA sequencing (VELCRO-IP RNA-seq), a versatile methodology to generate species-adapted ESs and to map specific mRNA regions across the transcriptome that preferentially associate with ESs. Application of VELCRO-IP RNA-seq to a mammalian ES, ES9S, identified a large array of transcripts that are selectively recruited to ribosomes via an ES. We further characterize a set of 5′ UTRs that facilitate cap-independent translation through ES9S-mediated ribosome binding. Thus, we present a technology for studying the enigmatic ESs of the ribosome, revealing their function in gene-specific translation.

## In Brief

Leppek et al. develop a pulldown technology employing chimeric yeast ribosomes, VELCRO-IP RNA-seq, to map interactions between ribosomal RNA (rRNA) and mRNAs genome-wide with positional precision. They find that expansion segments (ESs), the extended rRNA tentacles of the ribosome, specifically bind 5′ UTR elements to enable cap-independent translation of select mRNAs.

## Graphical Abstract

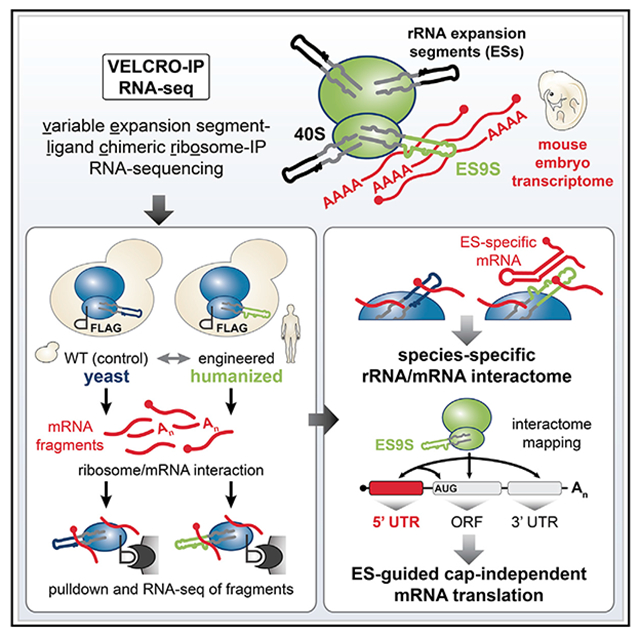

## INTRODUCTION

The ribosome is life’s most ancient molecular machine, with an RNA structural core that is universally shared across all species. However, a dramatic increase in its size has occurred during eukaryotic evolution. For example, the human ribosome is 1 MDa larger than the yeast ribosome, which in turn is another 1 MDa larger than the bacterial ribosome. This is largely because of the insertions of blocks of sequences called expansion segments (ESs) as they expand the eukaryotic ribosomal RNA (rRNA): the longest ESs are more than 700 nt in *Homo sapiens* (*H. sapiens*) and resemble flexible tentacles that extend from the ribosomal surface (Anger et al., 2013; Armache et al., 2010; Gerbi, 1996). Although ESs are generally found at the same relative location in the rRNAs of different eukaryotes, they can exhibit a striking degree of variability in their length and sequence both within and among species, including across tissue types (Kuo et al., 1996; Leffers and Andersen, 1993; Parks et al., 2018). ESs are located in rRNA regions of lower primary sequence conservation, which initially suggested that they are neutral mutations that do not interfere with essential rRNA functions in protein synthesis across all kingdoms (Gerbi, 1986).

However, the potential biological impact of ES variation on regulation of translation could be a critical facet in the understanding of the evolution of gene expression control and organismal development. We have previously discovered that the mammalian ES9S in 18S rRNA directly interacts with a 5′ untranslated region (UTR) RNA element in a *Homeobox* (*Hox*) transcript to promote translation initiation of the *Hox* mRNA in a transcript-specific manner. Specifically, a short P4 stem-loop of the *Hoxa9* internal ribosome entry site (IRES) -like element interacts with ES9S to promote cap-independent translation initiation (Leppek et al., 2020). It remains unanswered whether additional mRNAs genome-wide may also be recruited by ES9S and, more broadly, whether other ES variants across species or tissues may mediate currently unknown modes of translational control.

The major challenge in the functional investigation of ESs lies in that rRNAs are transcribed from ribosomal DNA (rDNA) loci that consist of hundreds of tandemly repeated units. The rDNA copy number varies among eukaryotic species, for example, ranging from a few hundred copies in most metazoans up to thousands of copies in wheat (Appels et al., 1980). Thus, for most higher eukaryotes, it has not been possible to experimentally manipulate rRNA to identify functions for a specific ES within the context of the assembled ribosome. Therefore, for the last several decades, the field has been limited in the understanding of ES function by the lack of a robust system to manipulate and investigate rRNA at the genetic and molecular levels.

Here, we report the development of VELCRO (variable expansion segment-ligand chimeric ribosome), a methodology to generate chimeric ribosomes in which the species-specific ES under investigation replaces its native counterpart on the yeast ribosome. Such chimeric ribosomes can be coupled modularly to a biochemical pulldown approach and RNA sequencing (VELCRO-immunoprecipitation [IP] RNA sequencing [RNA-seq]) to interrogate rRNA-mRNA interactions genome-wide. ES9S was chosen as a proof-of-principle ES variant of interest to develop the presented technology. By applying VELCRO-IP RNA-seq to mammalian ES9S, we discover an unexpected function of ES9S in gene regulation. This approach finds transcriptome-wide binding of specific mRNAs to ES9S through their 5′ UTRs, which enables cap-independent translation of the mRNA in a species-specific manner. These results highlight the role of the evolution of the ribosome ESs in guiding gene-specific translation and provide a technology broadly applicable to investigate enigmatic variations in rRNA.

### Design

We set out to explore a potential broader function of the ribosome ESs. Comprehensive methods to study ES function, or any rRNA functions beyond peptide bond formation, are lacking. This is because genetic manipulation of rDNA regions has not been possible for most higher eukaryotes due to the repetitive nature of hundreds of rDNA units spread across multiple chromosomal loci in metazoans (Romanova et al., 2006). Thus, a method that overcomes these limitations is required to pursue the question of specific mRNA recruitment to the ribosome via an ES. Such a method would enable broader inquiries into the function of ribosome ESs in general and across species-, tissue-, or individual-specific rDNA variants.

Baker’s yeast, *Saccharomyces cerevisiae* (*S. cerevisiae*), despite possessing a repetitive tandem array of rDNA units, contains a single rDNA locus in its genome. This locus has previously been deleted and can be complemented with an exogenous rDNA-expressing plasmid that enables genetic manipulation of ribosomes in yeast (Nemoto et al., 2010; Wai et al., 2000). This led us to envision a strategy in which the variable ES of interest could replace the native ES sequence of the yeast rRNA through a rDNA complementation approach. The importance of investigating ESs in the context of the full ribosome is particularly noteworthy. The study of individual ES RNA prepared *in vitro*, outside of the context of the full ribosome, would miss key maturation steps that occur during ribosome biogenesis *in vivo*, including critical cleavages, modifications, and chaperoned RNA folding.

**Figure 1.**
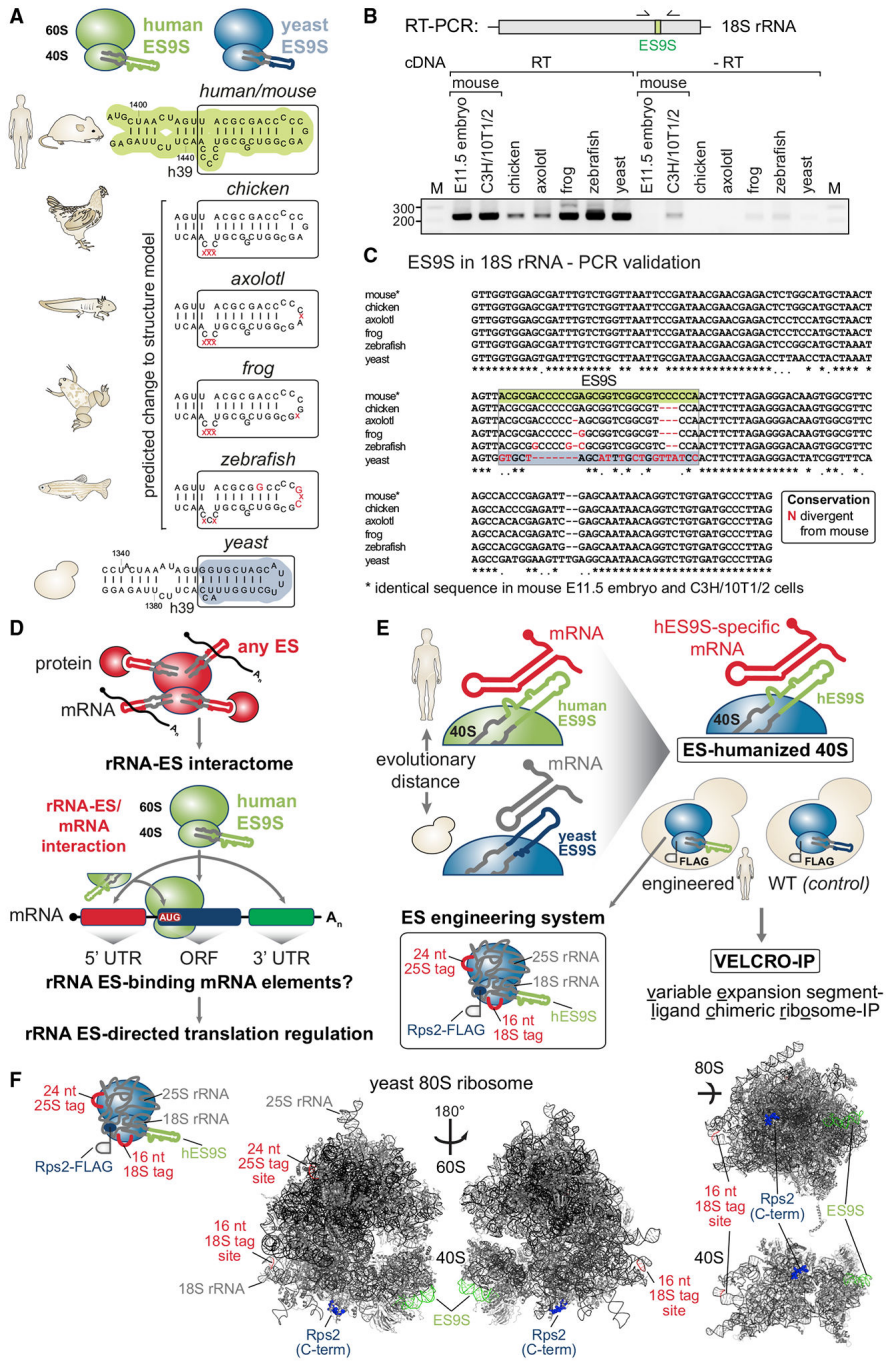
Confirmation of interspecies sequence variation of the ES9S 18S rRNA region (A) Secondary structure models of the human (*H. sapiens*) and baker’s yeast (*S. cerevisiae*) 18S rRNA region containing ES9S, highlighted in green and blue, respectively. Predicted structural changes in ES9S because of species-specific variation in sequence. Sequence divergence from the human/mouse ES9S are annotated in red. Secondary structure models of ES9S were predicted using Vienna RNAfold (http://rna.tbi.univie.ac.at) and visualized using VARNA (http://varna.lri.fr). See also Figure S1. (B) Schematic of the RT-PCR analysis of the ES9S region using cDNA generated from total RNA from six species (E11.5, stage E11.5 FVB mouse embryo; chicken, *Gallus gallus*; axolotl, *Ambystoma mexicanum*; frog, *Xenopus laevis*; zebrafish, *Danio rerio*; yeast, *Saccharomyces cerevisiae*) and primers specific for the 18S rRNA region containing ES9S (see Table S3). (C) Multiple sequence alignment of the variable ES9S region in highly conserved 18S rRNA. PCR product sequencing after RT-PCR spanning the ES9S region with the outer primers in (B) for six species confirms the annotated species-specific ES9S sequence. Nucleotides divergent from human/mouse ES9S are highlighted in red. (D) Concept of revealing extended rRNA ES interactions on the ribosome with mRNAs or proteins. This enables analysis of ES9S interactions, the ES of choice in this work, via the 40S ribosomal subunit with positional resolution to identify and map ES9S binding mRNA elements underlying unexplored ES-directed translation regulation. (E) Schematic of the VELCRO-IP (variable expansion segment-ligand chimeric ribosome-IP) approach to investigate ES-mediated translation regulation through mRNA interactions. Generating FLAG-tagged humanized ribosome strains that exclusively contain human ES9S in yeast 18S rRNA and tagged WT control yeast strains in parallel enables an ES engineering system that contains rRNA and protein tags and allows the manipulation of any ES. (F) Mapping of the components of the ES engineering system onto the cryoelectron microscopy (cryo-EM) structure of the yeast 80S and 40S ribosome (PDB: 4V6I). The sites of rRNA tag insertion,the last 10 amino acids of the C terminus of Rps2/uS5, and ES9S are highlighted according to the schematic representation.

**Figure 2.**
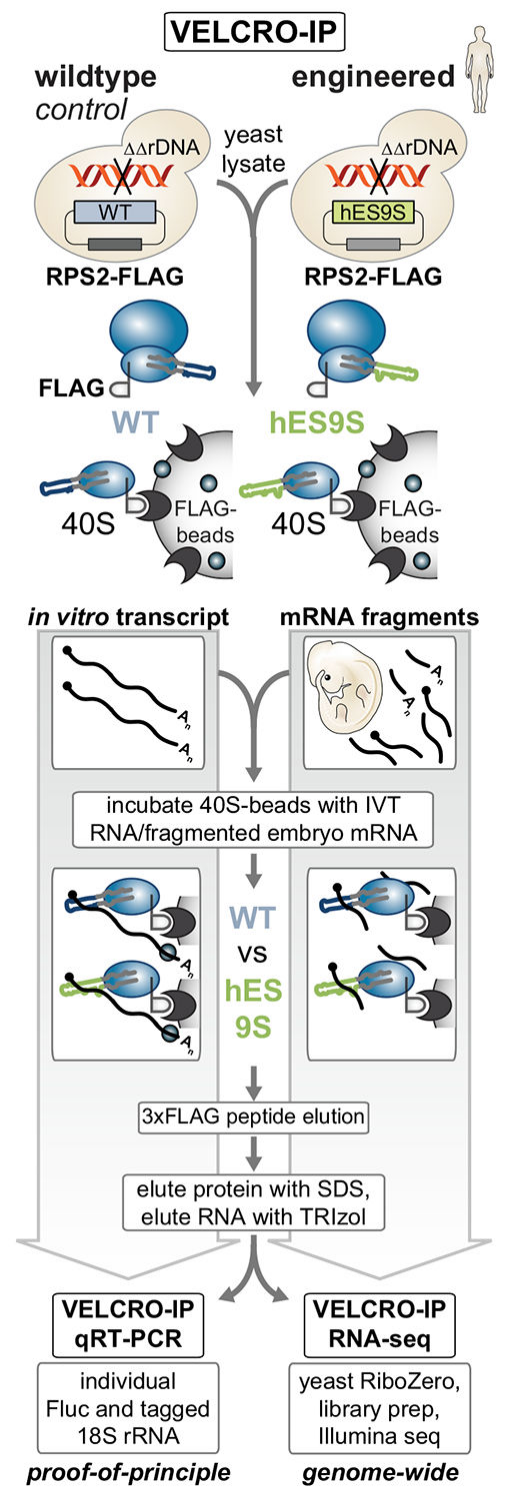
Development of VELCRO-IP RNA-seq to identify global ES-mRNA interactions Schematic representation of the VELCRO-IP approach. Yeast strains expressing chimeric (hES9S) orWT ribosomes are generated by rDNA complementation. The same strains also carry endogenously C-terminally FLAG-tagged RPS2/uS5. 40S ribosomal subunits from powderized lysates of each strain are isolated on FLAG agarose beads and washed. For VELCRO-IP qRT-PCR (proof of principle), *in vitro* transcripts (IVTs) (see Figure 3) are incubated with ribosome beads. Upon 3xFLAG peptide elution of 40S-RNA complexes, total RNA is eluted, and IVT RNA enrichment is determined by qRT-PCR specific for Fluc and the 18S rRNA tag. For VELCRO-IP RNA-seq (genome-wide), mRNAs from total RNA from stage E11.5 mouse embryos are purified and fragmented to 100–200 nt, and refolded RNA fragments are used as input for IP and FLAG elution of mRNA-ribosome complexes. After yeast rRNA depletion from eluted RNAs, ribosome-bound mRNA fragments are sequenced to identify hES9S-specific mouse mRNA elements.

**Figure 3.**
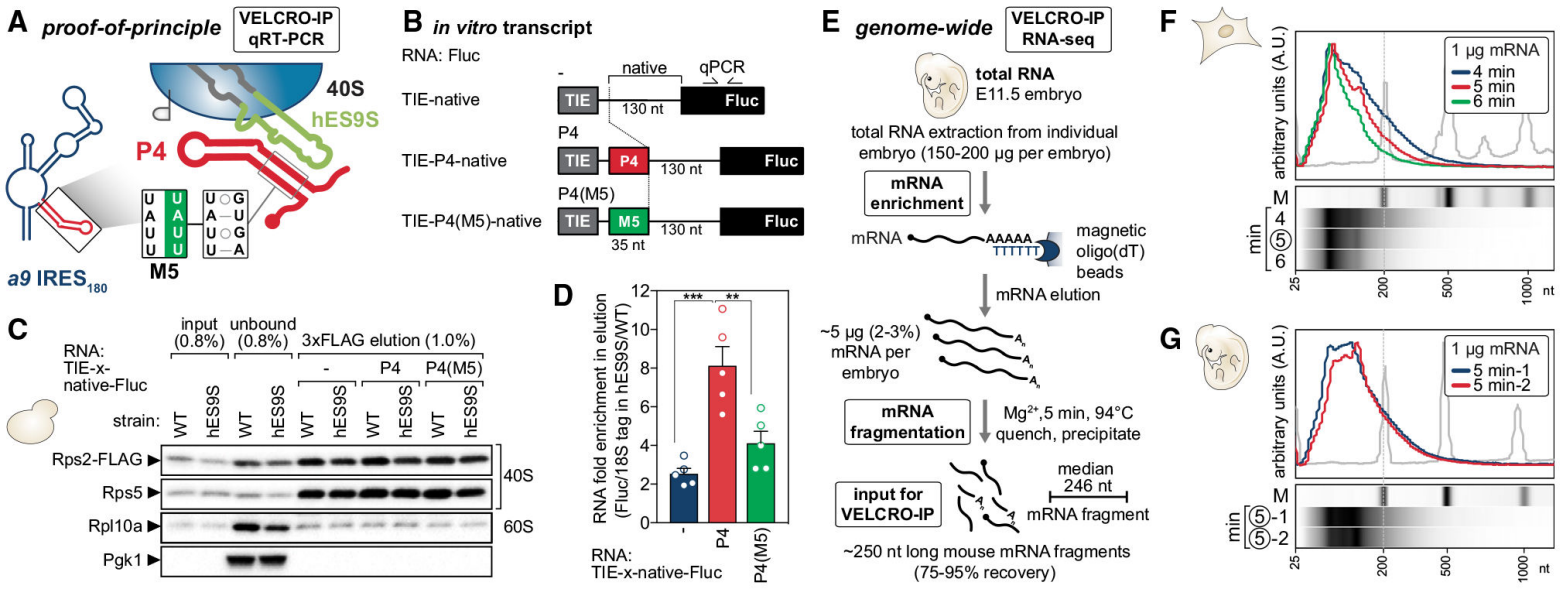
VELCRO-IP qRT-PCR serves as a proof of principle and mouse embryo mRNA fragmentation (A) VELCRO-IP qRT-PCR: a zoomed-in view on the interactions between hES9S and *Hoxa9* P4 stem-loop (Leppek et al., 2020) or other target 5′ UTRs that can be identified by VELCRO-IP. The 4-nt inactive P4 mutant M5 (P4(M5)) serves as a negative control. (B) IVTs of 475–510 nt in length contain the native spacer (–, negative control), P4-native (P4), or P4(M5)-native (P4(M5)) embedded in flanking constant regions (5′ TIE and 3′ Fluc ORF sequence) (see Leppek et al., 2020). The Fluc ORF portion can be used for qPCR amplification to compare the three RNA constructs. TIE, translation inhibitory element. (C) Western blot (WB) analysis of same volumes of lysate (input), unbound fraction, and 3xFLAG peptide-eluted protein from beads to monitor ribosome enrichment of tagged (Rps2-FLAG) and untagged (Rps5) 40S and 60S (Rpl10a) components in IVT RNA samples, in combination with WT and hES9S yeast ribosomes. Cytoplasmic enzyme Pgk1 served as a negative control. The fraction loaded of input, unbound, and elution samples is expressed as a percentage of the original lysate volume. A representative experiment of n = 5 is shown. (D) Analysis of total RNA in the 3xFLAG peptide elution by qRT-PCR using the same volumes of RNA per sample for the RT. Fluc transcript enrichment was assessed by normalizing Ct values to those of the respective 18S rRNA tag to control for ribosome-IP efficiency per sample. Respective hES9S samples were compared with WT samples to assess RNA fold enrichment of IVT RNAs. Average RNA fold enrichment ± SEM, n = 5. See also Figures S2E-S2G. (E) Schematic of embryo mRNA fragmentation for VELCRO-IP RNA-seq. Total RNA extraction of stage E11.5 mouse embryos yields 2%–3% of mRNA isolated on oligo(dT) beads. mRNA is fragmented with magnesium ions to a length of 100–200 nt, which overall recovers >75% of input mRNAs as fragments. (F) Fragmented mouse mRNAs from C3H10T1/2 cells in 1-μg aliquots at different time points of fragmentation (4, 5, and 6 min) were analyzed on an mRNA Pico Chip (Agilent) on a Bioanalyzer (Agilent). A zoomed-in view of the Bioanalyzer quantification (top) and virtual gel images (bottom) is shown. The marker (gray line, lane M) is overlaid for reference. See also Figures S3A-S3C. (G) Fragmented mouse mRNAs from stage E11.5 embryos in 1-μg aliquots fragmented for 5 min at 94°C from two independent repeats of embryo harvest, RNA isolation, mRNA purification, and fragmentation (1 and 2). This yields fragments of 100–200 nt. RNAs were analyzed as in (F). See also Figure S3C.

**Figure 4.**
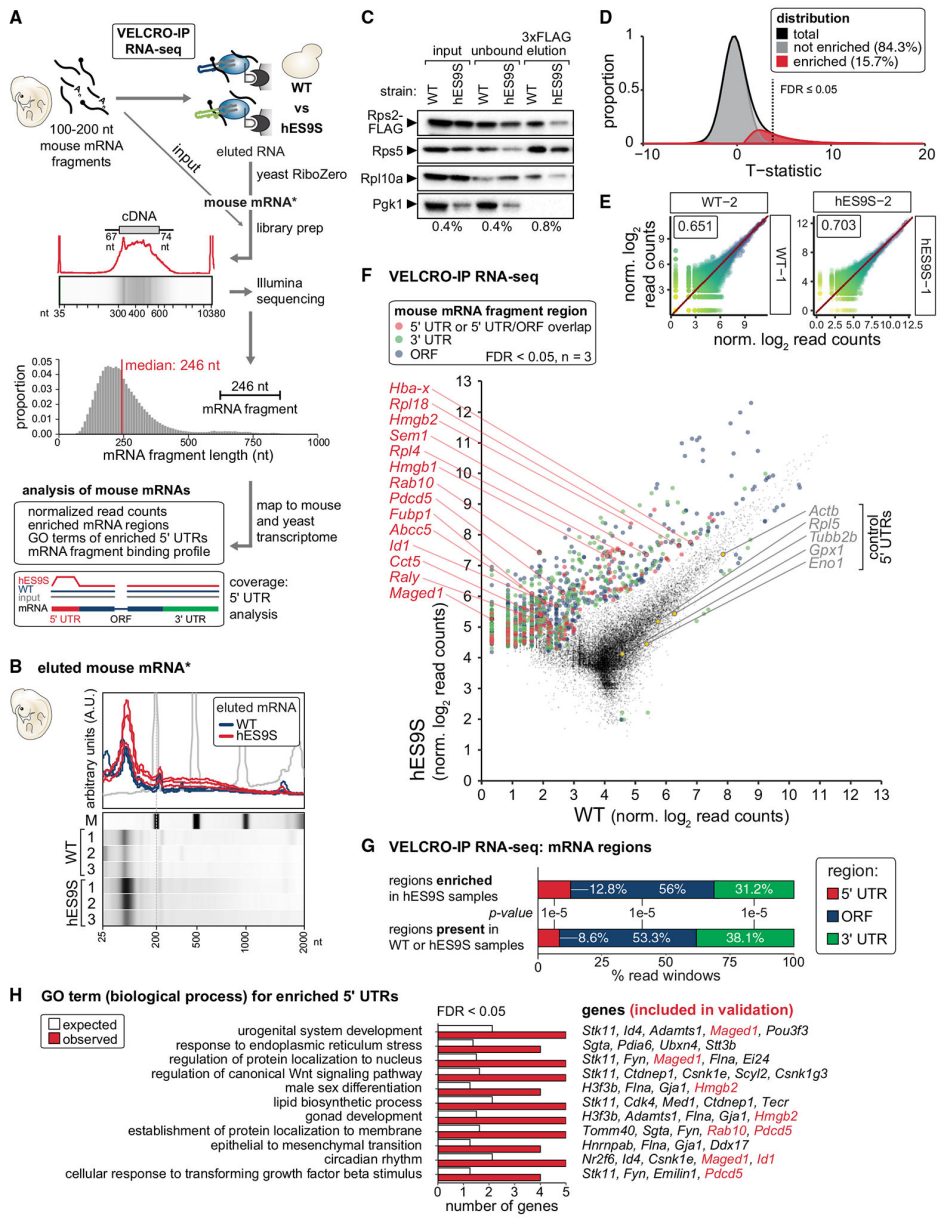
VELCRO-IP RNA-seq identifies global ES-mRNA interactions with positional resolution on mRNAs (A) For VELCRO-IP RNA-seq, mRNA was isolated from stage E11.5 mouse embryos, fragmented, and used as input. Eluted and yeast rRNA-depleted RNA obtains ribosome-bound mouse mRNA fragments for library preparation and Illumina sequencing, including the mRNA fragment input for reference. The distribution of mRNA fragment lengths for all sequenced libraries is plotted with a median fragment length of 246 nt. All reads were mapped to the mouse and yeast transcriptomes, and only reads exclusively mapping to mouse mRNAs were further analyzed. (B) Eluted and yeast rRNA-depleted mouse RNA from three independent replicates of WT and hES9S VELCRO-IP experiments were analyzed on an mRNA Pico Chip (Agilent) on a Bioanalyzer (Agilent) as in Figure 3F. See Figure S3D. (C) WB analysis as in Figure 3C to monitor efficient IP of 40S ribosomes after VELCRO-IP. A representative experiment of n = 3 is shown. (D) Kernel density of the distribution of t-statistics for the test of differential enrichment of mRNA fragments bound to hES9S versus WT ribosomes is plotted in black. Empirical estimates of the decomposition of the test statistics distribution to null and non-null tests are plotted in gray and red, respectively. The dotted line indicates local FDR of 0.05. (E) Comparison of individual VELCRO-IP RNA-seq samples (three replicate samples per hES9S and WT). Scatterplots of normalized log read counts, colored by expression level. Pearson correlation coefficients are shown in the top-right boxes. See Figure S4A. (F) RNA-seq results of independent replicates (n = 3) for each WT and hES9S sample. Normalized log read counts are presented for WT and hES9S-enriched mouse mRNA fragments. Fragments (FDR < 0.05) are colored according to the mRNA region to which they map (see legend): 5′ UTR or overlapping 5′ UTR/ORF (red), 3′ UTR (green), and ORF (blue). Mouse genes are labeled for which enriched fragments in the 5′ UTR and/or 5′ region of the ORF were identified and for which 5′ UTR validation experiments were performed. Five control 5′ UTRs are marked that are equally bound to both WT and hES9S 40S subunits and served as negative controls. See Figure S4B and Table S4. (G) Analysis of regions mapping to 5′ UTR, ORF, or 3′ UTR in hES9S-enriched samples compared with their presence in WT or hES9S samples, each n = 3, expressed as the percentage of total read windows identified. The indicated p value is calculated by a chi-square test. (H) Gene Ontology (GO) analysis for the biological process of 87 5′ UTR regions (FDR < 0.05, n = 3) enriched by hES9S. Displayed are the expected and observed frequency of genes for the significant terms (FDR < 0.05) (expressed mRNA regions were used as the background population; see STAR Methods for details of the thresholds used). See Figure S5 for GO terms of ORF, 3′ UTR, and full mRNA (all regions), as well as Table S5.

**Figure 5.**
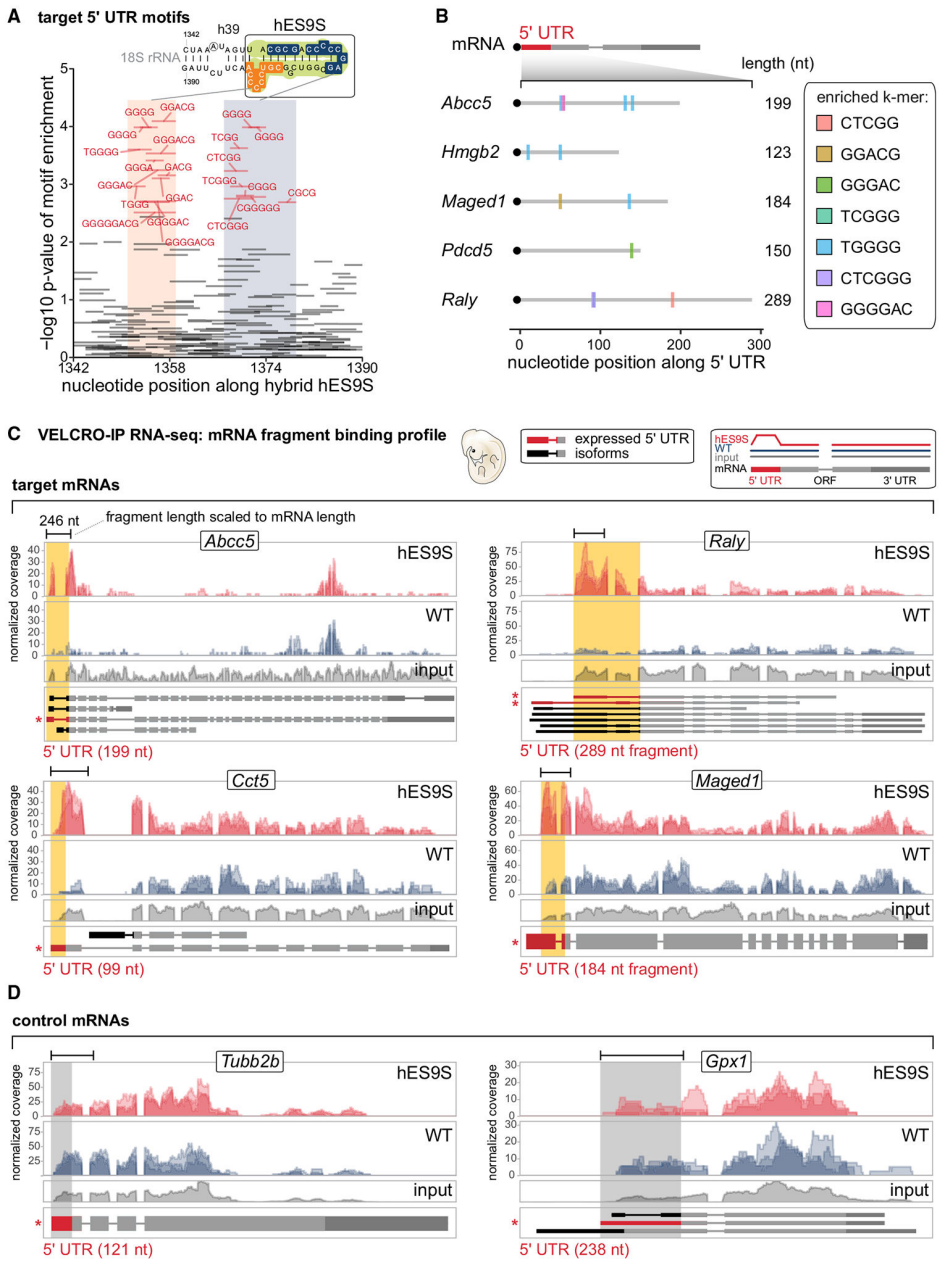
VELCRO-IP RNA-seq identifies hES9S-interacting 5′ UTRs with potential hES9S complementary and positional precision (A) Potential regions of canonical base-pairing between hES9S and hES9S-enriched mRNAs. The k-mers (4 ≤ k ≤ 8) in the reverse complement sequence of hES9S are plotted as short horizontal lines along the x axis. The y axis shows the Wilcoxon rank-sum test p values between counts of each k-mer across hES9S-enriched versus all 5′ UTR windows. Lines in red are significant k-mers with FDR ≤ 0.05. The colored bases in the inset hES9S structure indicate the bases included by significantly overrepresented k-mers mapping to two clusters in hES9S highlighted on the structure and shaded in the graph in orange and blue. (B) Selected individual examples of hES9S-enriched 5′ UTRs, with the overrepresented k-mers mapped onto the 5′ UTR. Highly hES9S-enriched 5′ UTR windows of *Abcc5*, *Hmgb2*, *Maged1*, *Pdcd5*, and *Raly* are plotted as lines, and each rectangular block indicates the positions of the significantly overrepresented k-mer, colored by each k-mer (k ≥ 5). (C) mRNA binding profile as coverage plots for four genes whose 5′ UTR-overlapping windows are significantly enriched in the hES9S over WT samples (FDR < 0.05, n = 3). Normalized per base coverage of individual biological replicate libraries for WT (blue) and hES9S (red) samples is plotted. All mRNA isoforms annotated in the ENSEMBL database are displayed below. Exon lengths are to scale, whereas intron lengths are pseudo-scaled. The read coverage of the input mRNA fragments (gray) is plotted for reference. 5′ UTR regions for the most likely expressed mRNA isoform in embryos (red) and the corresponding regions in the tracks (yellow) are shaded. The 5′ UTR region used for experimental validation corresponds to the asterisk-marked isoform. The mRNA fragment length for each gene is scaled according to the mRNA length for the individual genes presented. The mRNA fragment length, and thus the positional resolution of the coverage tracks, is approximately 100–200 nt. See Figure S6A. (D) Same analysis as in (C) was performed for two 5′ UTRs for which no enrichment of hES9S interaction over WT was found. 5′ UTR regions for the most likely expressed mRNA isoform in embryos (red) and the corresponding regions in the tracks (gray) are shaded. See Figure S6B.

**Figure 6.**
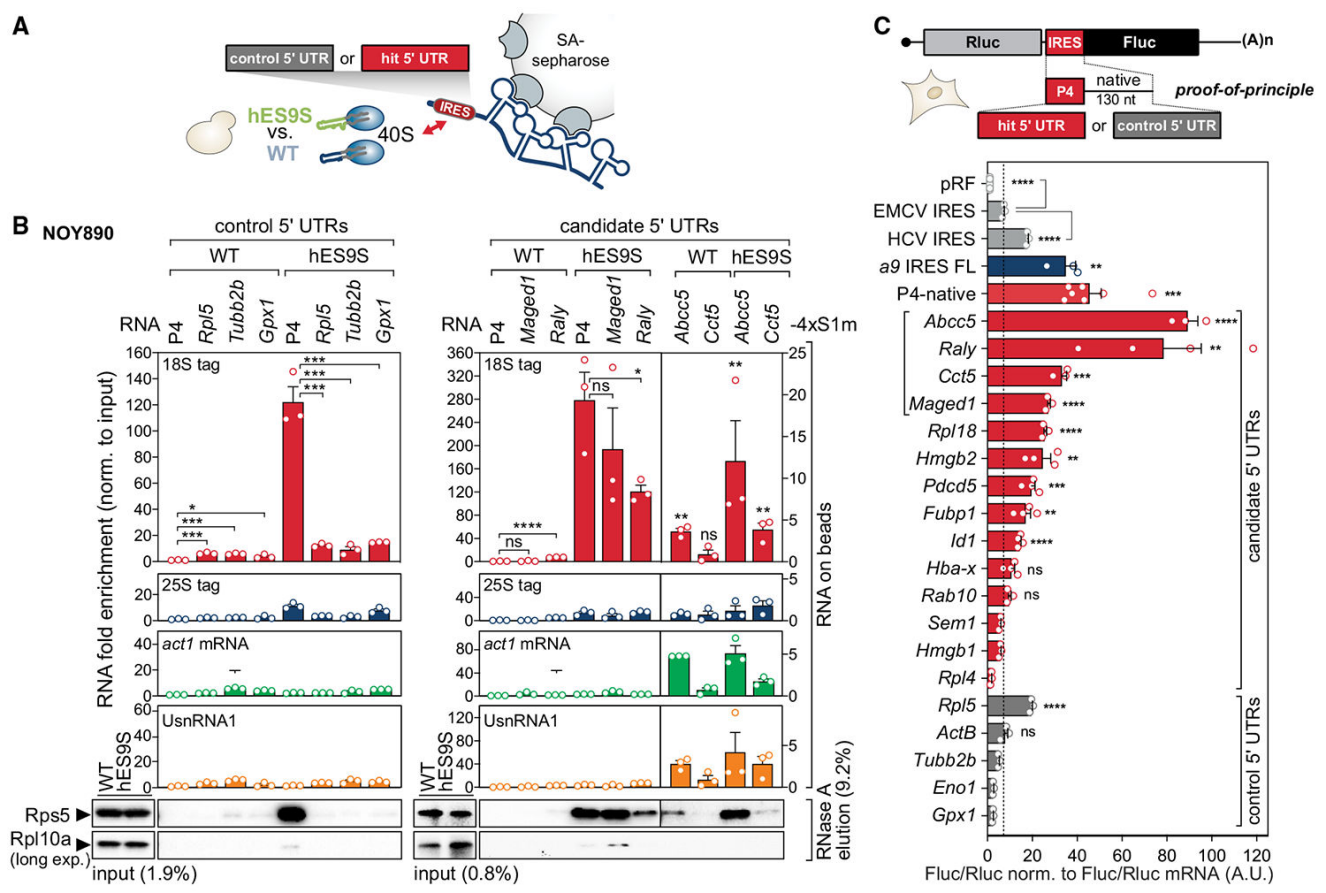
VELCRO-IP RNA-seq identifies hES9S-interacting 5′ UTRs with cap-independent translation initiation activity (A) Based on the analysis in Figures 5 and S6, full 5′ UTRs (as annotated in ENSEMBL) were experimentally validated. Schematic of the 4xS1m pulldown to probe the interactions of control and candidate 5′ UTR-4xS1m *in vitro*-transcribed RNAs with WT and hES9S yeast ribosomes. (B) 4xS1m pulldown of candidate 5′ UTR-4xS1m RNA with WT and hES9S yeast ribosomes for three control 5′ UTRs as negative controls and four candidate 5′ UTRs were tested alongside *Hoxa9* P4 as a positive control. After the formation of ribosome-RNA RNPs *in vitro*, beads are split in half for total RNA and protein. Ribosome-RNA RNP enrichment *in vitro* is monitored by qRT-PCR for tagged 18S and 25S rRNA and other RNA classes normalized to the input (RNA on beads) and by WB. Fold enrichment of RNAs was determined by qRT-PCR using the same volumes of eluted RNA and normalizing Ct values of each sample to their respective RNA input (WT or hES9S). Yeast *actin* (*act1*) mRNA and yeast UsnRNA1 serve as negative controls. WB analysis was performed for 40S and 60S subunit RPs of the same volumes of protein released from beads by RNase A. The fraction loaded of input and elution samples is expressed as a percentage of the original lysate volume. The P4-4xS1m/WT sample was used to normalize for RNA fold enrichment (set to 1). Average RNA fold enrichment, SEM, n = 3; ns, not significant; long exp., long exposure. See Figure S6C. (C) Bicistronic mRNA reporter genes containing no insert in the intergenic region (pRF, vector) and candidate or control 5′ UTRs were transiently transfected into mouse C3H10T1/2 cells. Cells were split in half for protein lysates for luciferase activity measurement and total RNA extraction for qRT-PCR analysis. Relative luciferase activity is expressed as a Fluc(IRES)/Rluc(cap-initiation) ratio normalized to respective Fluc/Rluc mRNA levels and expressed as average activity ± SEM, n = 3–8. pRF serves as negative control, the encephalomyocarditis virus (EMCV) and hepatitis C virus (HCV) IRESs serve as IRES controls, EMCV IRES activity was used as a cutoff, and the full-length (FL) *Hoxa9* IRES-like element and P4-native served as *Hoxa9* IRES-like references.

For the development of the VELCRO-IP method, the diversity in sequence and structure of ES9S in 18S rRNA, as a paradigm example, was first examined across species. We engineered chimeric ribosomes by humanizing yeast 18S rRNA exclusively in the distal part of ES9S (Figures 1 and S1) (Leppek et al., 2020). An endogenous FLAG tag was introduced to enable affinity purification of chimeric ribosomes, and the incorporation of chimeric, FLAG-tagged ribosomes into translating polysomes was verified (Figure S2). Furthermore, a pulldown method via the FLAG tag was established to selectively purify rRNA-mRNA interactions from an input pool of fragmented mouse embryo mRNAs (Figures 2, 3 and S3). Then, high-throughput RNA-seq was used to identify regions of embryonic mRNAs that interact with the humanized ES in a genome-wide fashion (Figure 4). By quantifying the enrichment of human ES9S compared with wild-type (WT) yeast ES9S across replicates, we ensured that the uncovered interactions are highly specific (Figures 4, 5, and S4). Importantly, a reverse pulldown approach orthogonally validated the discovered mRNA-ES interactions using the same yeast strains employed for VELCRO-IP (Figures 6 and S6). Altogether, VELCRO-IP RNA-seq offers a versatile, modular, and rigorous methodology to investigate variations in rRNA.

## RESULTS

### Engineering of yeast ribosomes with customized rRNA ESs for VELCRO-IP

When the secondary structures of 18S rRNAs for evolutionarily distant baker’s yeast (*S. cerevisiae*) (Armache et al., 2010) and human (*H. sapiens*) (Natchiar et al., 2017) are compared (Figures 1A and S1A-S1C), the basal stem region of helix h39 adjacent to ES9S is highly conserved, whereas the distal portion of ES9S is highly variable in length, structure, and sequence (Figures 1A-1C, boxed region). Even among vertebrate species that are more closely related, such as chicken (*Gallus gallus*), axolotl (*Ambystoma mexicanum*), frog (*Xenopus laevis*), and zebrafish (*Danio rerio*), nucleotide insertions and deletions in ES9S can affect ES9S structure (Figures 1C, S1D, and S1E). Their presence was confirmed by RT-PCR using cDNA from tissues of the respective species (Figures 1B and 1C).

The divergence in ES sequence is the prerequisite for and essence of VELCRO-IP. This method harnesses the interspecies variability in ESs to uncover the differential mRNA interactome of a defined ES and to functionally test the importance of ES sequences for species-specific mRNA binding and translation (Figures 1D and 1E). Thus, VELCRO-IP was designed to rely on the constant core of the ribosome, with all its exposed binding surfaces for proteins and RNAs and editing only one ES sequence at a time. This relies on several crucial strategies in terms of ribosome design: (1) employing yeast ribosomes as minimal ribosomes onto which evolutionarily distant metazoan ES sequences can be scarlessly transplanted; (2) carefully designing interspecies ES transplants according to rRNA structure, such that highly conserved constant regions are chosen as the edit site; (3) inserting RNA sequence tags into 18S and 25S rRNA to distinguish edited rRNA ribosomes from WT ribosomes for IP-enrichment analysis by qRT-PCR; (4) generating tagged WT-ribosome strains containing the yeast ES, along with chimeric ribosome strains for direct comparison; and (5) endogenously C-terminally FLAG-tagging a 40S ribosomal subunit protein, RPS2/uS5, in the rDNA deletion yeast strains to facilitate isolation of WT and chimeric 40S ribosomal subunits. This approach yields yeast ribosomes that contain 18S and 25S rRNA sequence tags, a Rps2-FLAG tag, and either a WT or a chimeric ES of choice (Figures 1E and 1F).

VELCRO-IP is designed to be applicable to any ES using the ribosome engineering system in yeast (Figures 1E and 1F). VELCRO-IP uses the yeast ribosome core to accomplish this, because yeast only has a single rDNA locus containing hundreds of tandemly repeated rDNA copies. The entire rDNA locus can be deleted and complemented with exogenous expression plasmids containing engineered rDNA sequences (Nemoto et al., 2010; Wai et al., 2000), which has been employed previously to study rRNA mutations (Venema et al., 1995). Such engineered humanized hybrid ribosomes for ES9S (Figures 1E and S1) contain humanized ES9S (hES9S) introduced scarlessly into the h39 stem region of yeast 18S rRNA that is highly conserved in sequence and structure (boxed region in Figure 1A). Mouse ES9S and human ES9S are 100% identical. Although we refer to chimeric ribosomes as hES9S, the *Hoxa9* 5′ UTR, as well as the transcriptome employed in this study, is of mouse origin.

It was crucial to design hybrid rRNAs according to RNA structure, only transplanting the most distal part of the foreign ES onto yeast 18S rRNA (Figures S1B and S1C). This complementary exchange of smaller regions is important, because deletion of large regions from most ESs, performed in yeast and *Tetrahymena thermophila*, can lead to ribosome biogenesis defects and to severe viability defects (Jeeninga et al., 1997; Ramesh and Woolford, 2016; Sweeney et al., 1994). Therefore, although ESs have been previously manipulated by large deletion or sequence exchange, it led to general defects in ribosome biogenesis. Such complete ES deletions preclude a more specific analysis of ES functions in translational control.

Next, unique sequence tags were introduced into both 18S and 25S rRNAs (Fujii et al., 2009) to quantitatively distinguish the humanized chimeric ribosomes from potentially remaining untagged WT ribosomes by qRT-PCR before ribosome purification (Figures 1E, 1F, and S2A-S2C). Because VELCRO-IP relies on the comparison of the interactomes of chimeric versus WT ribosomes, tagged but otherwise unmodified ribosomes were generated in parallel that retain yeast ES9S (referred to hereafter as WT). For tagged hES9S-ribosome-containing yeast strains, yeast cells that are induced to exclusively contain tagged hES9S ribosomes were confirmed to be viable and only showed a slight growth defect in comparison to tagged WT-rRNA-containing cells in a viability assay (Leppek et al., 2020).

This paved the way for the successful isolation of yeast strains after rDNA plasmid shuffling into the NOY890/Rps2-FLAG strain that solely contained plasmid-derived tagged hES9S or WT 18S rRNA ribosomes (Figures 1E and S2A) (Nemoto et al., 2010). Positive clones after shuffling were characterized by RT-PCR specific for the length difference of the ES9S sequence and the presence of the 18S rRNA tag (Figure S2B). Using qRT-PCR, the numbers of tagged and endogenous ribosomes pre sent after shuffling in these cells were also quantified (Figure S2C). The latter determined that only one endogenous plasmid-derived WT ribosome still remained per every 44 edited WT or per every 22 hES9S ribosomes. This untagged WT ribosome is thus minimal and irrelevant for the later detection of differentially enriched mRNAs. Although ribosomes had been affinity purified via MS2 RNA tags in rRNA in *E. coli* previously (Youngman and Green, 2005), we decided to tag a ribosomal protein (RP) for purification. This approach had been proven to be robust previously (Jan et al., 2014) and limited rRNA sequence manipulation to the ES region and rRNA tags. To this end, endogenous *RPS2/uS5* was C-terminally FLAG tagged, a technique previously used to successfully tag yeast ribosomes for isolation (Jan et al., 2014) (Figure S2A). Sucrose gradient fractionation of yeast lysates and western blot analysis confirmed that Rps2-FLAG protein is present in the heavy polysomes in both FLAG-tagged WT- and hES9S-ribosome strains (Figure S2D). This lack of difference in polysome profiles indicates no difference in translation rates between the strains. In addition, the comparison of hES9S and WT strains with and without the Rps2-FLAG indicated that another control 40S ribosomal subunit component, Rps5/uS7, is found normally incorporated into translating polysomes in both strains (Figure S2D). Rps2-FLAG is present in the same heavy polysome fractions as Rps5. FLAG-tagged RP incorporation into translating ribosomes is a prerequisite for isolation of mature ribosomes by VELCRO-IP. These strains could therefore next be used as a tool to study species-specific mRNA-ES interactions.

### VELCRO-IP employs purification of engineered humanized yeast ribosomes

With the straightforward generation of tagged chimeric ribosomes at hand, we next asked whether and which mammalian mRNAs in the transcriptome may recruit the 40S ribosome by binding to hES9S. To answer this question, VELCRO-IP was combined with a pulldown strategy, in which chimeric and WT yeast ribosomes are captured and used as bait to identify differentially bound mRNAs genome-wide (Figure 2). The modularity of this workflow allows the choice of not only the ES but also any tissue- or cellderived transcriptome that is relevant to a biological question.

As an mRNA input, either *in vitro*-transcribed RNA from plasmids encoding the *Hoxa9* 5′ UTR (proof of principle) or fragmented poly(A)-enriched mRNA from embryonic day (E) 11.5 mouse embryos (genome-wide) was used, as described later. For VELCRO-IP RNA-seq (Figure 2), fragmented mRNA from E11.5 mouse embryos were pooled and refolded in three steps of decreasing temperature to slowly reconstitute RNA structures such as short stem-loops. An input RNA sample was collected at this step for RNA-seq. A key design element that ensures the specificity of the detected hES9S-mRNA interaction in this protocol is the parallel generation and comparison of the interactomes of the WT and hES9S yeast ribosomes. This workflow can be performed in a day and is highly modular because it relies on sequential steps: (1) bead-based ribosome purification; (2) incubation with any pool of putatively interacting RNAs; (3) efficient, ribosome-specific 3xFLAG peptide elution; and (4) quantitative analysis of the eluted RNA.

### VELCRO-IP qRT-PCR enables interrogation of variant ES-specific ribosome-mRNA interactions

We have previously shown that hES9S ribosomes are sufficient to reconstitute binding to the *Hoxa9* 5′ UTR, particularly to the 35-nt P4 stem-loop in the *Hoxa9* IRES-like RNA element, which highlights the ES specificity of this mRNA-rRNA binding event (Figure 3A) (Leppek et al., 2020). In a proof-of-principle experiment, we tested whether hES9S ribosomes could specifically interact with an *in vitro*-transcribed minimal reporter mRNA containing P4. This positive control is called TIE-P4-native, because it also contains the 5′ translation inhibitory element (TIE) present within the endogenous *Hoxa9* 5′ UTR and inhibits cap-dependent translation (Xue et al., 2015). This construct also has a native spacer sequence between the P4 and the start codon, which is required for translation initiation. The *in vitro* transcript with only the TIE and the native spacer sequence without P4 thus serves as a negative control (TIE-native). Another negative control is a 4-nt inactivating mutation within P4, termed P4(M5), that disrupts the ES9S interaction (TIE-P4(M5)-native). All three RNA constructs were positioned upstream of the Fluc open reading frame (ORF) sequence. The 3′ Fluc ORF sequence allows comparable Fluc-specific qPCR quantification (Figures 3B, S2E, and S2F). For the pulldown, ~500-nt-long *in vitro* transcripts of TIE-native, TIE-P4-native, or TIE-P4(M5)-native RNAs were generated. First, the FLAG pulldown of ribosome-mRNA complexes from yeast lysates was performed to enrich for 40S ribosomal subunits. For this, WT- and hES9S-rRNA-expressing NOY890/RPS2-FLAG strains were harvested in the mid-log phase of actively translating cells. For ribosome isolation, Rps2-FLAG-tagged 40S ribosomes were immunoprecipitated from lysates on anti-FLAG M2 affinity agarose gel. Previous experience had shown that agarose gel beads are advantageous over magnetic beads to cleanly isolate ribosomes (Simsek et al., 2017) with higher affinity. This first purification step yields ribosome beads of washed yeast 40S ribosomal subunits bound via Rps2-FLAG before incubation with an RNA input source. Refolded RNAs were added to and incubated with WT and hES9S ribosomes on FLAG beads for IP. Bound ribosome-mRNA fragment complexes were washed and eluted off the anti-FLAG beads using competitive 3xFLAG peptide elution. The IP and elution efficiency was monitored by protein analysis using WB and total RNA extraction for qRT-PCR analysis. The analysis of specific protein and RNA enrichment in eluates demonstrated that (1) VELCRO-IP cleanly isolates tagged 40S ribosomal subunits (Figure 3C) and (2) in comparison to WT, yeast hES9S ribosomes enrich P4-containing transcripts about 4-fold more than P4-less TIE-native RNA (Figures 3D and S2G). The clear reduction in hES9S-ribosome binding to the inactive P4(M5) mutant highlights the specificity and sensitivity of the VELCRO-IP approach (Figures 3D and S2G).

### VELCRO-IP RNA-seq uses mRNA fragments to map hES9S-interacting mRNA regions

The VELCRO-IP qRT-PCR results for control *in vitro* transcripts paved the way for a genome-wide version of the ribosome pulldown experiment, VELCRO-IP RNA-seq, that uses fragmented mouse embryo mRNAs to identify mRNA regions that may preferentially rely on hES9S for ribosome binding (Figure 2, right). To gain positional information of bound mRNA regions that an ES preferentially interacts with—for example, within 5′ UTR, ORF, or 3′ UTR (Figure 1D)—random fragments of the input mRNA in the size range of 100–200 nt were employed (Figure 3E). To generate a pool of endogenous mouse embryo mRNAs as a physiological source of RNA, stage E11.5 mouse embryos were harvested individually, which yielded 150–200 μg of total RNA per embryo. Purified embryo mRNA was fragmented to a 100- to 200-nt range by hydrolysis with magnesium ions and heat. Fragmentation was optimized for time (0–10 min) and mRNA input amount (250 ng, 500 ng, and 1 μg of mRNA) by monitoring RNA size using urea-PAGE (Figure S3A) and by Bioanalyzer analysis (Figures 3F, 3G, S3B, and S3C). mRNA fragmentation was first optimized using mRNA from C3H10T1/2 mesenchymal cells, which performed identically to purified stage E11.5 embryo mRNA (Figures 3G and S3C). Immediate precipitation recovered 75%–95% of input mRNA as mRNA fragments. VELCRO-IP RNA-seq uses 10 μg of fragmented mRNAs as input. After yeast ribosome-IP, ribosomes were incubated with fragmented and refolded mouse embryo mRNA and ribosome-mRNA complexes were eluted with 3xFLAG peptide (Figure 4A). Eluted RNAs mainly consist of yeast rRNA, which were depleted to increase the representation of mouse mRNA fragments in the final RNA-seq library. An overall enrichment of mouse mRNA fragments in hES9S samples compared with WT controls was detected by Bioanalyzer analysis (Figures 4B and S3D). The IP and elution efficiency were confirmed by WB analysis (Figure 4C). The sequencing libraries were prepared from yeast rRNA-depleted eluted RNAs, using randomly primed reverse transcription and incorporating unique molecular tags before amplification. The cDNA libraries were sequenced using the high-throughput Illumina platform (Figure 4A).

### VELCRO-IP RNA-seq identifies ES9S-interacting mRNA elements genome-wide

Three replicate VELCRO-IP RNA-seq experiments were performed and sequenced for WT- and hES9S-ribosome interactions. The final median fragment length observed in the sequencing library was 246 nt (Figure 4A). Sequencing reads mapping to mouse transcripts in 200-nt windows were counted across the genome. We detected 18,989 windows across 2,610 genes with sufficient coverage for statistical tests of differential enrichment of mRNA fragments bound to hES9S over WT yeast ribosomes. Using empirical modeling of the test statistic distribution, 15.7% of the 18,989 regions were estimated to be differentially enriched and thus have binding dependency on hES9S (Figure 4D). The three independent repeats for WT and hES9S samples were highly reproducible (Figures 4E and S4A). At a false discovery rate (FDR) of 5%, 1,491 regions over 460 genes could be confidently classified as strong candidates for further analysis (Figures 4F and S4B; Table S4). This indicates a pervasive, hES9S-dependent binding of selective mRNA regions to ribosomes transcriptome-wide. Moderate overrepresentation of the hES9S-enriched windows in 5′ UTR over ORF and 3′ UTR regions of the mRNA was observed (~1.7-fold, p < 1 x 10^−5^) (Figures 4F and 4G). Among the group of 460 genes whose mRNAs preferentially bound to humanized ribosomes (Table S4), 87 genes were identified whose enriched regions overlap with their 5′ UTRs. They are enriched for Gene Ontology (GO) terms involving developmental and differentiation processes, such as regulation of Wnt signaling pathways, gonad development, and urogenital system development (Figure 4H; Table S5). Another interesting category is that of circadian rhythm, whose biology frequently involves translational control for temporal expression patterns, such as melanoma antigen-encoding gene D1 (*Maged1*) and inhibitor of DNA binding 1 (*Id1*). GO term enrichment analysis for coding sequence (CDS), 3′ UTR, or all regions together revealed other diverse types of functional annotations, such as cell cycle, DNA damage responses, or muscle contraction (Figure S5; Table S5). These data together suggest that hES9S-bound mRNAs may be involved in post-transcriptional regulation of multiple important functional pathways, especially in mammalian embryonic development.

### Sequence complementarity in mRNA-hES9S interactions

Although canonical Watson-Crick pairing between mRNA and rRNA nucleotides is a key aspect of translation initiation in viruses or prokaryotes (Shine and Dalgarno, 1974; Steitz and Jakes, 1975), it is not thought be a broadly used mechanism in eukaryotes, with only a few examples known thus far (Dresios et al., 2006; Martin et al., 2016; Weingarten-Gabbay et al., 2016). We asked whether there may be a role for canonical complementarity in the interaction between mRNAs and hES9S in rRNA. We searched for all possible short substrings of length k (k-mers, 4 ≤ k ≤ 8) that may be overrepresented in the hES9S-enriched mRNA 5′ UTR regions along the reverse complement sequence to hES9S as an indication for potential canonical base-pairing interactions between hES9S and 5′ UTRs. Two clusters of k-mers complementary to hES9S were found to be significantly overrepresented (Figure 5A). Examining these k-mers in individual hES9S-enriched 5′ UTR regions, many examples of hES9S-bound 5′ UTRs with multiple significant k-mers were found within each 5′ UTR (Figure 5B). Thus, canonical Watson-Crick pairing may be one mode of interaction between hES9S and transcripts.

### hES9S binding profiles and hES9S-interacting mRNA elements that mediate cap-independent translation

An orthogonal approach was employed to validate the interaction of the 5′ UTRs with chimeric hES9S ribosomes for four of the identified 5′ UTR candidates: ATP binding cassette subfamily C member 5 (*Abcc5*), heterogeneous nuclear ribonucleoprotein (hnRNP) associated with lethal yellow (*Raly*), chaperonin containing TCP1 subunit 5 (*Cct5*), and *Maged1* (Figure 5C). Comparing the read coverage in hES9S and WT samples across the expressed mRNA (input) revealed specific enrichment of the 5′ UTR and/or the region overlapping the first exon for these mRNAs in hES9S samples (Figures 5C and S6A). Three control 5′ UTRs were also included—*Rpl5*, tubulin beta 2B class IIb (*Tubb2b*), and glutathione peroxidase 1 (*Gpx1*)—that are not selectively bound by hES9S (Figures 5D and S6B). They were selected based on their estimated negative predictive values and confidence intervals (Figures 4D, and 4F). In a reverse approach to VELCRO-IP, 4xS1m pulldown experiments were performed as established previously (Leppek and Stoecklin, 2014; Leppek et al., 2013), using the 5′ UTRs as RNA bait for WT and hES9S ribosomes that use yeast cell lysates as input (Figure 6A). Compared with the positive control, *Hoxa9* P4, there was no enrichment for any control 5′ UTRs, including *Rpl5*, to hES9S ribosomes. In contrast, significant enrichment was observed of all candidate 5′ UTRs identified in the VELCRO-IP RNA-seq experiments (Figures 6B and S6C). *Maged1* and Raly bind to the hES9S 40S ribosomal subunit in the same range as the *Hoxa9* P4 element. These results demonstrate the high specificity of the genome-wide VELCRO-IP RNA-seq analysis.

Beyond the functional correlation of selective hES9S-dependent binding to ribosomes, we asked whether hES9S-enriched 5′ UTRs mediate cap-independent translation initiation activity, similar to that of the *Hoxa9* 5′ UTR. Thus, full-length mouse 5′ UTRs were tested in bicistronic mRNA reporters (Figure 6C). Nine of fourteen candidate 5′ UTRs exhibit cap-independent activities higher than the viral encephalomyocarditis virus (EMCV) IRES, which served as a reference and positive control. Enriched hES9S reverse complement k-mers are found in the 5′ UTRs that exhibit cap-independent translation reporter activities (k ≥ 5 shown in *Abcc5*, *Hmgb2*, *Maged1*, *Pdcd5*, and *Raly* 5′ UTRs) (Figure 5B). These results suggest that canonical base-pairing with hES9S may be important for recruitment of mRNAs to the ribosome and to promote cap-independent translation. To assess the likelihood of cap-independent activity in 5′ UTRs without hES9S interaction, five control 5′ UTRs that clearly were not enriched in hES9S over WT in the genome-wide VELCRO-IP RNA-seq data were also tested (Figures 4F, 5D, 6C, and S6B). These results suggested that the specificity of the mRNA-hES9S interaction as determined by VELCRO-IP RNA-seq functionally selected for cap-independent activity.

In terms of the confirmed hES9S target mRNAs, *Maged1* is known to be important for brain and bone formation (Bertrand et al., 2004; Liu et al., 2015), including possible regulation of homeodomain transcription factors such as *Dlx5* and *Msx2* (Masuda et al., 2001). *Raly* encodes an RNA binding protein, which has been implicated in early pre-implantation embryonic development (Michaud et al., 1993). These data thus identified critical physiological regulators that specifically recruit ribosomes for cap-independent translation through hES9S. Prior comparative analysis of *Maged1* expression during brain and embryonic development has revealed a discrepancy between mRNA and protein expression levels, suggesting that its expression levels are regulated at the post-transcriptional level (Bertrand et al., 2004).

Altogether, the VELCRO-IP RNA-seq approach represents a powerful tool to reveal how ribosome-mediated control of gene regulation is achieved at the molecular level in a genome-wide manner. In combination with orthogonal mRNA reporter and pulldown assays for validation, this methodology represents a targeted strategy to further identify mRNAs that directly bind to any ES on the ribosome.

## DISCUSSION

The existence of rRNA sequence variation across species, as well as within a species, is becoming increasingly apparent with advances in genomics technologies. For example, variant rDNA operons can be selectively expressed in prokaryotes under stress (Kurylo et al., 2018; Song et al., 2019). During zebrafish development, distinct maternal-type and somatic-type ribosomes that vary in ES sequence can be detected (Locati et al., 2017). Analysis of transcriptomics data has suggested differential expression of variant rDNA species across human populations, as well as across tissues within an individual (Parks et al., 2018). The variable ESs in eukaryotes therefore reflect a playground for evolutionary diversity among rRNA sequences.

Yet beyond their existence, the question of whether inter- and intra-species rDNA variation in ESs is functional has remained largely unanswered, especially in mammals. Methodological challenges in designing genetics approaches for repetitive sequences limited previous studies to observational investigations. Here, we developed VELCRO-IP, which directly addressed ES function using a combination of yeast genetics and biochemical approaches. The hES9S-mRNA interactome data revealed the genome-wide role of ES-mediated mRNA recruitment to the ribosome that promotes cap-independent translation initiation.

rRNA-mRNA interaction is a classic paradigm for translation initiation in prokaryotes, in which the Shine-Dalgarno leader sequence in mRNAs base-pairs with the 16S rRNA 3′ end to designate translation start sites (Shine and Dalgarno, 1974; Steitz and Jakes, 1975). In eukaryotes, one example of mRNA-rRNA interaction is found between the purine-rich sequence in the histone H4 mRNA coding region and helix h16 of the 18S rRNA, whose base-pairing tethers the 40S ribosome to the start codon (Martin et al., 2016). However, these mRNAs contact conserved rRNA segments rather than ESs. Beyond a few such direct mRNA-rRNA binding examples in eukaryotes for which a role in translation regulation has been suggested (Dresios et al., 2006; Martin et al., 2016; Weingarten-Gabbay et al., 2016), no clear evidence for the transcriptome-wide use of direct mRNA-rRNA binding as a widespread mechanism of translation initiation has been demonstrated for any eukaryotic species. Interestingly, signatures of canonical base-pairing interactions were found between hES9S and its target mRNAs, suggesting a potential mechanistic importance for sequence complementarity. Although this study focused on the 5′ UTR binding sites to ES9S based on the observed 5′ UTR enrichment, many strong interactions mapping to coding regions and the 3′ UTR were also identified, potentially broadening the scope of contributions from interactions made by different parts of the mRNA. In the future, it will be interesting to use VELCRO-IP to address whether sequence-specific recruitment of mRNAs to the ribosome may also be employed by other ESs.

We foresee numerous applications of VELCRO-IP in probing the effects of rRNA ESs on translation regulation, beyond the interspecies differences in ES sequence reported here. For example, it can be used to probe the potential functional consequences of rDNA variants across the human population. Furthermore, it need not be limited to the mRNA interactome: VELCRO-IP could be adapted to investigate the ES-bound proteome by coupling it to mass spectrometry. The function of ESs does not need to be exclusive to mRNA binding and translation initiation. For example, ES27L in yeast 28S rRNA acts as a scaffold to bind a methionine amino peptidase enzyme to control translation fidelity (Fujii et al., 2018; Knorretal.,2019; Wildetal.,2020). Mammalian ribosomes, beyond the classical core RPs, also interact with hundreds of additional ribosome-associated proteins (RAPs) to form the ribointeractome (Simseket al., 2017). It will be interesting to see whether recruitment of a RAP to the ribosome depends on variation in ESs and thus endow the ribosome with organism- or tissue-specific functions.

Altogether, the presented ribosome engineering approach provides an elegant and robust solution to address ribosome ES function by identifying ES interactions with *cis*-regulatory mRNA elements or RAPs. We envision that this methodology will lead to a more precise understanding of rRNA function in gene regulation in other translation-coupled cellular processes.

### Limitations

Users need to be aware of a couple of limitations to the VELCRO-IP strategy. First, for many yeast ESs, their complete deletion greatly reduces the level of the edited rRNA because of biogenesis defects (Ramesh and Woolford, 2016). Thus, the length of the exchanged sequence may be crucial. Incorporating longer replacement ES sequences may be challenging if this leads to ribosome biogenesis defects extreme enough to cause lethality.

Second, one cannot exclude that additional ES binding transcripts may rely on more elaborate structures or co-factors only present within an *in vivo* setting. VELCRO-IP is thus not sensitive to potential interactions that may require other cellular components such as adaptor proteins or RNA *trans*-acting factors, possible differential cellular RNA folding, or long-range interactions. In addition to missing some interactions that may require the endogenous cellular context, non-specific interactions can occur between RNA and protein complexes in solution. Traditional methods of RNA binding protein (RBP) bound target RNA identification via IP can be prone to post-lysis *in vitro* association of RBPs with spurious targets or reorganization of native ribonucleoproteins (RNPs) that are dynamic and thus highly sensitive to experimental conditions (Mili and Steitz, 2004). This notion highlights the need for critical and careful functional validation of the specific interaction of enriched mRNAs found by VELCRO-IP RNA-seq.

If feasible, an *in vivo* crosslinking approach may address potentially missed or artificial interactions. This strategy remains challenging to implement for rRNA ESs. This notion largely results from the lack of efficient and robust RNARNA crosslinking methods. Sequence-specific psoralen derivatives only capture interactions with *trans*-pyrimidine configurations (Calvet and Pederson, 1979; Cimino et al., 1985), which may be especially problematic given the high GC content of many ESs. In addition to the narrow sequence specificity, psoralen crosslinking is hardly reversible and inefficient for lowly abundant RNA species like mRNAs. Thus, an *in vivo* crosslinking approach is currently impractical to be generally applicable for most potential rRNA ES-mRNA interactions. The strength of the presented method lies in its general applicability and its highly specific enrichment readout.

## STAR★METHODS

### RESOURCE AVAILABILITY

#### Lead contact

Further information and requests for resources and reagents should be directed to and will be fulfilled by the Lead Contact, Maria Barna (mbarna@stanford.edu).

#### Materials availability

All plasmids and yeast strains generated in this study are available upon request and will be fulfilled by the Lead Contact, Maria Barna (mbarna@stanford.edu).

#### Data and code availability

RNA sequencing data from VELCRO-IP RNA-seq experiments are available in Table S4. The accession number for the RNA-seq data reported in this paper is Gene Expression Omnibus (GEO): GSE141382.

### EXPERIMENTAL MODEL AND SUBJECT DETAILS

#### Cell Culture and Transfection

C3H/10T1/2 (ATCC: CCL-226) cells were cultured in Dulbecco’s Modified Eagle’s Medium (DMEM, GIBCO, 11965–118) containing 2 mM L-glutamine, supplemented with 10% fetal calf serum (EMD Millipore, TMS-013-B), 100 U/ml penicillin and 0.1 mg/ml streptomycin (EmbryoMax ES Cell Qualified Penicillin-Streptomycin Solution 100X; EMD Millipore, TMS-AB2-C or GIBCO, 15140–122) at 37°C in 5% CO_2−_-buffered incubators. ~0.6 X 10^6^ C3H/10T1/2 cells were seeded per well in 12-well dishes and transfected the following day with 0.8-1.6 μg of plasmid using 4 μL Lipofectamine 2000 (Invitrogen, 11668-019) and Opti-MEM (GIBCO, 11058-021) according to the manufacturer’s instructions in serum-free and antibiotic-free DMEM. The medium was changed to regular DMEM 4-6 hours after transfection and cells were collected 24 hours post-transfection.

#### Mice

Mice were housed under a 12 h light/dark cycle with free access to food and water. FVB/NJ (Stock# 001800) mice were purchased from the Jackson Laboratory (Bar Harbor, ME, USA) and used as wild-type. Pregnant FVB females, 3-8 months of age, were euthanized at E11.5, the uterus was dissected and embryos were taken out and placed into 1x PBS (GIBCO, 14190-250). Embryos were individually collected in either TRIzol (Invitrogen, 15596) and lysed by pipetting for total RNA isolation or collected in 2 mL safe-lock tubes (Eppendorf) in 1x PBS, supernatant was removed and embryos were snap frozen in liquid nitrogen. For lysates, embryo pellets were homogenized by cryo-milling after addition of a 2.5 or 5 mm steel bead using a tissue lyser (QIAgen TissueLyser II) at 25 Hz for 15 seconds 3-6 times, and the powder was either processed directly or snap frozen in liquid nitrogen and stored at −80°C. All animal work was performed in accordance with protocols approved by Stanford University’s Administrative Panel on Laboratory Animal Care.

#### Yeast Strains and Transformation

Yeast plasmids and strains (*Saccharomyces cerevisiae*) used in this paper are listed in Tables S1 and S2, respectively. Yeast strains were grown in YPD medium (10 g/L yeast extract, 20 g/L peptone, and 20 g/L glucose), YPAD medium (10 g/L yeast extract, 20 g/L peptone, 40 mg/L adenine sulfate, and 20 g/L glucose), or Synthetic Dextrose (SD) medium (6.7 g/L yeast nitrogen base, 20 g/L glucose, 1.6 g/L amino acids drop out mix (Complete Supplement Mixture, CSM, Sunrise Science Products)). All yeast strains were cultured at 30°C, unless specified otherwise. Cells were harvested in mid-log phase growth (OD_600_ = ~0.8). Plasmid transformation of yeast cells was performed using mid-log phase cells grown in YPD, YPAD, or SD medium and standard lithium acetatemediated transformation of 1 μg DNA and selection of transformants on SD plates of appropriate amino acids drop-out for 2-3 days at 30°C was performed.

The rDNA mutant strains were produced from the genomic rDNA deletion strain (KAY488 (NOY890)) (Nemoto et al., 2010), complemented rDNA with an exogenous plasmid, pRDN-hyg (*RDNA*^*hyg*^ *URA3*) (Nemoto et al., 2010; Wai et al., 2000), which was exchanged by plasmid shuffling to pNOY373 (*RDNA LEU2*) or derivatives containing human ES9S and 18S and 25S rRNA tags. To remove the pRDN-hyg plasmid, strains were negatively selected against the *URA3* marker gene using 1 mg/mL of 5-Fluoroorotic Acid (5-FOA) (Fisher Scientific, F10501-5.0) in SD-plates, which is processed to a toxic product by the Ura3 enzyme. To monitor rRNA processing, 5′ end processing of endogenous and tagged 18S and 25S rRNA were analyzed by qRT-PCR using pre-mature rRNAspecific or total rRNA primers (Fujii et al., 2009). Total RNA was extracted according to the manufacturer’s instructions (MasterPure Yeast RNA Purification Kit, Epicenter, MPY03100). Successful plasmid shuffling was confirmed by total RNA extraction and qRT-PCR for rRNA tags, as well as by plasmid miniprep and RT-PCR specific for the ES9S region and the 18S rRNA tag.

C-terminally FLAG-tagged RPS2/uS5 strains were generated in the KAY488 (NOY890) strain by transforming 1 μg of a linear DNA template with a Kanamycin resistance cassette and 40 nt of homology arms to the target site. Selection was performed on a YPAD plate containing 200 mg/L of Geneticin (GIBCO, 11811-031). Subsequently, rRNA-tagged WT and hES9S strains were generated by plasmid shuffling into this strain.

### METHOD DETAILS

#### Plasmid Construction

The following plasmids have been described previously: pSP73 (p2008) and pSP73-4xS1m (p2880) (Leppek and Stoecklin, 2014) were kindly provided by Georg Stoecklin; pSP73-4xS1m(MCS) (Leppek et al., 2020); pRF (Rluc-Fluc bicistronic; Rluc, Renilla luciferase; Fluc, Firefly luciferase reporter genes, driven by the SV40 promoter) and pRF-HCV and -EMCV (Yoon et al., 2006) were kindly provided by Davide Ruggero (UCSF); pRF derivatives containing *Hox* 5′ UTR elements and pGL3-FLB-TIE-FL containing IRES-like elements (Leppek et al., 2020; Xue et al., 2015).

In order to generate the series of bicistronic Rluc-IRES-Fluc pRF plasmids containing candidate 5′ UTRs from VELCRO-IP RNA-seq, full 5′ UTRs for all tested 5′ UTR-candidates and controls were either amplified from cDNA derived from E11.5 mouse mRNA reverse transcribed using SuperScript III and IV (Invitrogen, 18080044, 18090010) or synthesized (Twist Bioscience, San Francisco, USA) and inserted into the EcoRI/NcoI-sites of the bicistronic pRF vector (Yoon et al., 2006) by Gibson assembly using the NEBuilder HiFi DNA Assembly Master Mix (NEB, E2621S). Sequences were based off the ENSEMBL database (Zerbino et al., 2018) and expression profiles in input RNA-seq data. Derivatives of the plasmid pSP73-4xS1m(MCS) (Leppek et al., 2020) were generated by PCRamplifying 5′ UTR sequences from pRF plasmids using AccuPrime Pfx DNA Polymerase (Thermo, Invitrogen, 12344024). pSP73– 4xS1m(MCS) and derivatives can then be linearized at the EcoRI site downstream of the 4xS1m aptamers for run-off *in vitro* transcription.

For pNOY373-18S/25S-tag, into the yeast plasmid derivatives of pNOY373, we inserted rRNA tag sequences (Leppek et al., 2020), a 16-nt tag into 18S rRNA (Beltrame et al., 1994) and a 24-nt tag into 25S rRNA (Musters et al., 1989), for RT-PCR and qRT-PCR analysis. In a second step, the yeast ES9S was exchanged for the human ES9S in pNOY373-18S/25S-tag, which were generated by overlap extension PCR and were subsequently introduced into SacII-MluI-sites of pNOY373-18S/25S-tag, respectively. A list of all plasmids and primer sequences used are provided in Tables S1 and S3, respectively. All oligonucleotides were purchased from IDT. Mutations, cloning boundaries and coding sequences in all plasmids were verified by DNA sequencing (QuintaraBio).

#### Luciferase Activity Assay after Plasmid Transfection

Transiently transfected C3H/10T1/2 cells in 12-well plates were washed twice with 1x PBS (GIBCO, 14190-250) and collected by trypsinization 24 hours post-transfection for luciferase activity assays. Half the cells were used for assaying luciferase activity using the Dual-Luciferase Reporter Assay System (Promega, E1980) to measure Firefly (Fluc) and Renilla (Rluc) luciferase activities, the other half was collected in TRIzol (Invitrogen, 15596) for total RNA extraction and normalization to mRNA levels by qRT-qPCR (see qRT-qPCR section). For luciferase assays, cells were lysed in 60 μl of 1x passive lysis buffer of the Dual-Luciferase Reporter Assay System (Promega, E1980) and directly assayed or frozen at −20°C. After thawing, cell debris and nuclei were removed by centrifugation for 1 min at 13,000 rpm. 20 μl of supernatant was assayed for luciferase activity in technical replicates by mixing with 50 μl of Dual-Luciferase Reporter Assay System substrates. Fluc and Rluc activities were measured on a GloMax-Multi (Promega) plate reader. Luciferase reporter activity is expressed as a ratio between Fluc and Rluc which was normalized to the ratio of Fluc to Rluc mRNA levels for bicistronic pRF constructs to verify the integrity of the bicistronic mRNA construct. Each experiment was performed in three independent replicates at minimum. Statistical analysis was performed using unpaired two-tailed Student’s t test.

#### Quantitative reverse transcriptase PCR (qRT-PCR) Analysis

Cells transfected with pRF constructs were collected in 500 μL TRIzol (Invitrogen, 15596). Total RNA was isolated from the aqueous phase using RNA PureLink columns (Thermo Scientific, Ambion, 12183018) and treated with TURBO DNase (Ambion, AM2238) twice, followed by a second RNA PureLink column purification to remove plasmid DNA. For quantitative reverse transcriptase PCR (qRT-PCR) analysis, cDNA was synthesized from 100-200 ng of total RNA using iScript Supermix (Bio-Rad, 1708840) containing random hexamer primers, according to the manufacturer’s instructions. PCR reactions were assembled in 384-well plates using 2.5 μL of a 1:4-1:5 dilution of a cDNA reaction, 300 nM of target-specific primer mix and the SsoAdvanced SYBR Green supermix (Bio-Rad, 1725270) in a final volume of 10 μl per well. SYBR green detection qPCR was performed on a CFX384 machine (Bio-Rad). Data was analyzed and converted to relative RNA quantity manually or using CFX manager (Bio-Rad). Gene-specific qPCR primer sequences used for detection of mRNAs and rRNAs are given in Table S3.

#### *In vitro* RNP affinity purification via 4xS1m-aptamers

The 4xS1m-pulldown of RNP complexes was performed similar to as previously reported (Leppek and Stoecklin, 2014). RNAs were synthesized by *in vitro* transcription: RNA elements were fused to 4xS1m aptamers by cloning 5′ UTR amplicons into the BglII/EcoRV sites of pSP73-4xS1m(MCS). 4xS1m alone served as negative control RNA. Since amplification of the highly structured 4xS1m tag by PCR is problematic, linearized pSP73 plasmids served as DNA templates. Up to 20 mg template plasmid was linearized at the EcoRIsite downstream of the 4xS1m sequence in a 50 μL reaction for 6 hours or overnight, purified with the QIAquick PCR Purification Kit (QIAgen) and used as DNA templates for run-off *in vitro* transcription using MEGAscript SP6 kit (Ambion, AM1330). A 40 μl transcription reaction contained 8 μg linear DNA template, 4 μM of each NTP (Ambion), 4 μL/ 400 U MEGAscript SP6 RNA polymerase (Ambion) and 1x SP6 MEGAscript Transcription Buffer (Ambion). After incubation for 4-6 hours at 37°C, the DNA was digested by addition of 2 μL/4 U Turbo DNase (Ambion, AM2238) for 15 min at 37°C. Synthesized RNA was purified by gel filtration using prepacked G-50 Mini Quick Spin Sephadex RNA columns (Roche, 11814427001) according to the manufacturer’s instructions, and RNA concentration and quality was determined by Nanodrop and 4% urea-PAGE, respectively. One reaction typically yielded 50-200 μg of RNA.

For all steps in the pulldown experiments, 1.5 mL DNA/RNA LoBind tubes (Eppendorf) were used to reduce unspecific binding. Per sample, 100 μl 50% slurry of Streptavidin Sepharose High Performance (GE Healthcare) beads were washed three times with 0.5-1 mL of SA-RNP lysis buffer (20 mM Tris-HCl (pH 7.5, Ambion, AM9850G, and Ambion, AM9855G), 150 mM NaCl (Ambion, AM9759), mM MgCl_2_ (Ambion, AM9530G), 2 mM DTT (Ambion, 10197777001), and 1 tablet/10 mL Mini Complete Protease Inhibitors, EDTA-free (Sigma-Aldrich, Roche, 11836170001) in nuclease-free water (Thermo Fisher, Invitrogen, 10977023). At each step, beads were gently pelleted at 500 rpm (~20 x g) for 1 min at 4°C. ~30 μg of the *in vitro* transcribed 4xS1m or 5′ UTR-4xS1m RNAs per sample for pulldown from mouse or embryo powder for protein analysis or 2.5-7.5 μg of the *in vitro* transcribed RNAs per sample for pulldown of ribosomes from yeast was renatured in 50 μl SA-RNP lysis buffer by heating at 56°C for 5 min, 10 min at 37°C, and incubation at room temperature for several minutes to refold RNA structures. The RNA was added to the 100 μl SA Sepharose slurry together with 1 μl RNasin Plus RNase inhibitor (40 U/μL, Promega, N261A). 10 μl of the supernatant was saved for extraction of input RNA using TRIzol (Invitrogen, 15596), 2.5 μl of the supernatant (input RNA) was saved for urea-PAGE analysis, and 20 μL for an input protein sample. The mixture was incubated at 4°C for 2-3 hours under rotation to permit binding of the RNA to the column. Then, beads were sedimented and 2.5 μl of the supernatant (unbound RNA) was saved for urea-PAGE analysis, while the remaining supernatant was discarded. Input and unbound RNA samples were compared side by side by 4% polyacrylamide (Ambion)/0.5x TBE (Sigma)/urea (Sigma) gel electrophoresis and SYBR Gold (10,000x, Thermo Fisher, Invitrogen, S11494) staining in 0.5x TBE to assess the efficiency of RNA coupling.

For analysis of RNA-associated proteins and RNA from yeast cells, mid-log phase cells from a 1 L SD-LEU medium culture was harvested as described in the yeast section, washed once with water, and the cell pellet was split into 16 equal aliquots into 2 mL safe-lock tubes. The yeast pellets were then snap frozen in liquid nitrogen, homogenized by cryomilling after addition of a 2.5 mm steel bead using a tissue lyser (QIAgen TissueLyser II) at 25 Hz for 30 s 3–6 times, or until the tissue was powderized, and the powder was either processed directly or stored at −80°C. The frozen homogenate of one aliquot (~300 mg) was solubilized by the addition of 100 ml ice-cold RNP lysis buffer per sample and allowed to thaw for 5 min at room temperature or until thawed. Cell debris was removed by centrifugation for 5 min at 17.000 x g at 4°C, resulting in a supernatant of ~500 μl. Yeast samples were centrifuged again for 10 min at 17.000 x g at 4°C to remove remaining cell debris. The protein concentration in the extract was determined by Nanodrop to be ~25-70 mg/ml.

Next, the extract (~500 μl) was pre-cleared by addition of 25 μl of a 50% slurry of Avidin Agarose (Thermo Pierce) beads, 100 μl of a 50% slurry of SA Sepharose beads, and 5 μL RNasin (Promega), and tumbling for 2 hours at 4°C. Beads were collected and discarded, and the pre-cleared lysate was supplemented with 2 μl of RNasin Plus (Promega), added onto the freshly prepared, RNAcoupled SA Sepharose matrix, and incubated at 4°C for 2-3 hours under rotation to form RNP complexes. Beads were rinsed once and washed 3 times for 2-5 min with 1 mL SA-RNP wash buffer (20 mM Tris-HCl (pH 7.5), 300 mM NaCl, 5 mM MgCl_2_, 2 mM DTT, and 1 tablet/50 mL Complete Protease Inhibitors, EDTA-free (Roche) in nuclease-free water).

For qRT-PCR analysis of RNA and WB analysis of proteins from yeast cells, elution was performed as follows. After the last wash, beads were transferred to a fresh tube and resuspended in 500 μL SA-RNP lysis buffer. 250 μL were saved and used for TRIzol extraction of bound RNA according to the manufacturer’s instructions. 15 μg GlycoBlue (Ambion, LSAM9516) was added to the RNA prior to precipitation. RNA-bound proteins were eluted from the rest 250 μL of beads by addition of 2 μg RNase A (Invitrogen, AM2271, 1 μg/μL) in 30 μl Low Salt Buffer and rotation for 20 min at 4°C. The RNase A eluate was recovered, supplemented with SDS sample buffer and 8 μl of the eluate was analyzed by SDS-PAGE and WB. After RNase A elution, the beads were extracted with 30 μl 2x SDS sample buffer, 10 μl of which were analyzed by SDS-PAGE and WB. The fraction loaded of input and elution samples is expressed as percentage of the original lysate volume. For qualitative assessment of binding and elution efficiencies, an RNA fraction at each step was analyzed by 4% polyacrylamide/0.5x TBE/urea gel electrophoresis and SYBR Gold staining. For qRT-PCR analysis following RNA-IP, a fixed volume of 1:100 diluted RNA extracted from IP and input samples was used for reverse transcription. Each sample was normalized to the 18S-tag Ct values for that respective sample to control for ribosome-IP efficiency.

#### Western Blot Analysis and Antibodies

Proteins were resolved on 4%–20% polyacrylamide gradient Tris-glycine gels SDS-PAGE gels (Biorad, 567-1095, 456-1096) and transferred onto 0.2 μm pore size PVDF membranes (Biorad) using the semi-dry Trans-Blot Turbo system (Biorad, 170-4273). Membranes were then blocked in 1x PBS-0.1% Tween-20 containing 5% non-fat milk powder for 1 hour, incubated with antibodies diluted in the same solution for 1 hour at room temperature or overnight at 4°C, and washed four times for 5 min in 1x PBS-0.1% Tween-20, incubated with secondary antibodies for 1 hour in 1x PBS-0.1% Tween-20 and washed four times for 15 min in 1x PBS-0.1% Tween-20. Horseradish peroxidase-coupled secondary antibodies (anti-mouse and anti-rabbit, GE Healthcare; antirat, Jackson Immunoresearch) in combination with Clarity Western ECL Substrate (Biorad, 170-5061) and imaging on a ChemiDoc MP (Biorad, 17001402) were used for detection. Antibodies were diluted in 1x PBS-0.1% Tween-20 at 1:1000 dilution either in 5% BSA (w/v) or 5% non-fat milk. The following primary antibodies were used for western blot analysis: mouse monoclonal anti-FLAG (Sigma-Aldrich, M2, F3165), anti-PGK1 (Thermo-Fisher, Novex, 459250); rabbit polyclonal anti-RPL10A/uL1 (yeast: Santa Cruz, sc-100827), anti-RPS5/uS7 (Abcam, ab58345). Rabbit polyclonal anti-RPL10A antibody was kindly provided by Mary Ann Handel (The Jackson Laboratory, Bar Harbor, ME, USA).

#### Sucrose Gradient Fractionation Analysis in Yeast

For sucrose gradient fractionation of yeast cell lysates, the protocol as in Jan et al. (2014) was used with the following adjustments. Stationary yeast cultures of cell expressing WT or hES9S rRNA in the NOY890-WT or NOY890-RPS2-FLAG background were diluted to OD_600_ = 0.05 in 250 mL SD-LEU drop-out media and grown at 30°C. At mid-log phase (OD_600_ = 0.5-0.8), Cycloheximide (CHX) (Sigma Aldrich, C7698-1G) at 100 μg/ml was added into the medium and the culture was incubated for 5 min at 30°C shaking, prior to harvest omitting a water wash. Pellets were snap frozen in liquid nitrogen in 2 mL tubes. A cell pellet of a 250 mL culture was used per polysome gradient. Cell pellets were powderized by cryomilling after addition of a 2.5 mm steel bead using a tissue lyser (QIAgen TissueLyser II) 3 times at 25 Hz for 30 s, and the powder was processed directly. Frozen cell powder of a 250 mL culture was solubilized with 200 μL polysome lysis buffer (20 mM Tris-HCl pH 8.0 (Ambion, AM9855G), 140 mM KCl (Ambion, AM9640G), 1.5 mM MgCl_2_ (Ambion, AM9530G), 1 mM DTT (Ambion, 10197777001), 8% glycerol (Sigma-Aldrich, G5516), 1% Triton X-100 (Sigma-Aldrich, T8787), 100 μg/ml CHX (Sigma-Aldrich, C7698-1G), 100 U/mlSUPERase In RNase Inhibitor (Ambion, AM2694), 25 U/ml TurboDNase (Ambion, AM2238), Complete Protease Inhibitor EDTA-free (Sigma-Aldrich, Roche, 11836170001) in nuclease-free water (Thermo Fisher, Invitrogen, 10977023)) and vortexed. After lysis for 30 min on a rotator at 4°C, nuclei and cell debris were removed by two consecutive centrifugations (5,000 g, 5 min at 4°C, followed by 10,000 rpm, 10 min, at 4°C). Total RNA concentrations in cleared lysates were measured using a Nanodrop UV spectrophotometer (Thermo Fisher) and RNA-normalized amounts of lysates in 250 μL volume were layered onto a linear sucrose gradient (10%–45% sucrose (Fisher Scientific, S5-12) (w/v), 20 mM Tris-HCl, pH 8.0, 140 mM KCl, 5 mM MgCl_2_, 0.5 mM DTT, 100 μg/ml CHX) in nuclease-free water and centrifuged in a SW41Ti rotor (Beckman Coulter) for 2.5 hours at 40,000 rpm at 4°C. Typically, 750-1000 μg RNA was used for each sucrose gradient fractionation experiment. Fractions were collected by the Density Gradient Fraction System (Brandel, BR-188)with continuous A_260_ measurement. After collection of polysome fractions in 2 mL safe-lock tubes (Eppendorf), all fractions were individually precipitated using the Proteoextract Protein Precipitation Kit (EMD Milipore, Calbiochem, 539180-1KIT). For each 600 μL fraction, 450 μL precipitant 1 was added and incubated at −20°C for at least 1-3 hours. 10% of precipitated fractions were resolved in 26-well, 4%–20% SDS-PAGE gels (Biorad, 567-1095, 456-1096).

#### VELCRO-IP RNA-seq

The FLAG-pulldown of ribosome-mRNA complexes was performed the same way as for 4xS1m-mediated pulldowns from yeast, stated above. To enrich 40S ribosomal subunits, NOY890 strains that contain endogenously FLAG-tagged RPS2/uS5 at the C terminus were subjected to plasmid shuffling, as described in the yeast section, to generate tagged WT and hES9S rRNA expressing cells. Two individually isolated clones were used per strain. Cells of a 500 mL culture in SD-LEU medium were harvested when they reached mid-log phase (OD_600_ = ~0.8-1.0). 2x 250 mL pellets were washed once with water, cells were collected in a 1.5 mL tube and fash frozen in liquid nitrogen. For lysate preparation and to ensure scalability, 250 mL pellets were powderized in liquid nitrogen using a mortar and pestle and stored at −80°C. Addition of EDTA or puromycin to the lysis buffer to split ribosomal subunits was not needed. For ribosome isolation, RPS2-FLAG tagged 40S ribosomes were immuno-precipitated from lysates on anti-FLAG M2 affinity agarose gel. Previous experience had shown that agarose gel beads are advantageous over magnetic beads to cleanly isolate ribosomes (Simsek et al., 2017) with higher affinity. This first purification step yields a ribosome beads-resin of washed 40S ribosomal subunits bound via Rps2-FLAG before incubation with an RNA input source.

For the proof-of-principle pulldown experiment using 475-510 nt long *in vitro* transcripts of native, P4-native or M5-native RNAs flanked by TIE and Fluc sequences, DNA templates were amplified from monocistronic pGL3 plasmids using a SP6-flanked forward primer and Fluc-specific reverse primer (KL414/KL415) and the MEGAscript SP6 kit (Ambion, AM1330), as described in the 4xS1m pulldown section. RNA yields of 250 μg were obtained, quality was assessed by native 4%–20% TBE PAGE and by SYBR Gold staining. For the FLAG-pulldown experiments as described in more detail below, 5 or 7.5 μg aliquots of each *in vitro* transcript was refolded in 100 μL lysis buffer (20 mM Tris-HCl (pH 7.5, Ambion, AM9850G, and Ambion, AM9855G), 150 mM NaCl (Ambion, AM9759), 1.5 mM MgCl_2_ (Ambion, AM9530G), 2 mM DTT (Ambion, 10197777001), and 1 tablet/10 mL Mini Complete Protease Inhibitors, EDTA-free (Sigma-Aldrich, Roche, 11836170001 in nuclease-free water), and added to 50-100 μL ribosome-coupled anti-FLAG M2 agarose beads and 1 μL RNasin (Promega) per reaction. Samples were rotated for 2 hours at 4°C, rinsed once and washed 3 times with 500 μL-1 mL wash buffer (20 mM Tris-HCl (pH 7.5), 300 mM NaCl, 5 mM MgCl_2_, 2 mM DTT and 1 tablet/50 mL Complete Protease Inhibitors, EDTA-free (Roche) in nuclease-free water) with rotation, before competitive 3xFLAG peptide elution in 150 μL lysis buffer for 1 hour at 4°C with rotation, as stated below. 5% of the elution was used for protein analysis by WB, and 95% was subjected to TRIzol total RNA extraction and qRT-PCR analysis.

In order to generate a pool of endogenous mouse embryo mRNAs as RNA input for the ribosome-IP, up to 10 stage 11.5 embryos per FVB female were harvested as described in the mouse section, individually collected in 2 mL Eppendorf tubes, washed once with 1x PBS (GIBCO, 14190-250), and lyzed in 1 mL TRIzol (Invitrogen, 15596) by pipetting and vortexing, and addition of another 800 μL TRIzol. Embryo lysates were stored at −80°C until total RNA extraction. From each embryo, 150-200 μg total RNA was obtained. From total RNA, poly(A) mRNA was isolated on oligo(dT) beads using the Oligotex mRNA Mini Kit (QIAgen, 70022) or Poly(A) Purist MAG kit (Invitrogen, AM1922) according to the manufacturer’s instructions, which yielded ~5 μg mRNA (2%–3%) of 150-200 μg total RNA per embryo. Purified embryo mRNA was fragmented to 100-200 nt RNA fragments by magnesium-buffer based degradation using the NEBNext Magnesium RNA Fragmentation Module (NEB, E6150S). Fragmentation was optimized for time and RNA input amount monitoring RNA size using the mRNA Pico Chip (Agilent) on a Bioanalyzer (Agilent), and by 8% denaturing urea-PAGE and SYBR Gold staining. mRNA fragmentation was initially optimized using mRNA isolated from mouse C3H10T1/2 mesenchymal cells instead of embryo tissue and the yield of purified mRNA was identical from different source material. We tested input mRNA amounts of 250 ng, 500 ng and 1 μg mRNA over a time course of 0-10 min, since the manufacturer’s protocol only indicated use for up to 250 ng mRNA. Fragmentation of 1 μg mRNA aliquots for 5 min at 94°C in 1x Fragmentation Buffer (NEB) was optimal to obtain a pool of 100-200 nt fragments. Reactions were quenched on ice and by addition of 1x Stop Solution (NEB). Immediate isopropanol-based precipitation recovered 75%–95% of input mRNA as mRNA fragments in water.

For FLAG-pulldown of FLAG-tagged yeast 40S, powderized yeast lysates of a 250 mL culture per three samples were dissolved in 500 μL lysis buffer (20 mM Tris-HCl (pH 7.5, Ambion, AM9850G, and Ambion, AM9855G), 150 mM NaCl (Ambion, AM9759), 1.5 mM MgCl_2_ (Ambion, AM9530G), 2 mM DTT (Ambion, 10197777001), and 1 tablet/10 mL Mini Complete Protease Inhibitors, EDTA-free (Sigma-Aldrich, Roche, 11836170001 in nuclease-free water) and the tube was washed with another 200 μL lysis buffer. Lysates were cleared by centrifugation for 5 min at 17,000 g at 4°C and 2 min at 17,000 g at 4°C, and 800 μL lysate was recovered. RPS2-FLAG tagged 40S ribosomes were immuno-precipitated by addition of 50 μL washed anti-FLAG M2 affinity agarose gel (Sigma Aldrich, A2220-5mL) and 5 μL RNasin Plus (Promega) per sample to 800 μL lysate and 1.5-2 hours of rotation at 4°C. Beads were washed 3 times with 500 μL lysis buffer and bound ribosomes were resuspended by addition of 200 μL lysis buffer. 10 μg fragmented mRNA from E11.5 FVB embryos in 40 μL per sample were pooled for 6 samples. 5 μL was saved as an input RNA sample for sequencing. Pooled mRNA was refolded in lysis buffer in a total volume of 600 μL as described in the 4xS1m pulldown section and used as input for 6 samples. 10 μg refolded RNA in 100 μL was added to 100 μL ribosome-coupled 50% beads, 3 μL RNasin (Promega) and 100 μL lysis buffer for a total volume of 300 μL for IP by rotation for 2 hours at 4°C. Bound ribosome-mRNA fragment complexes were rinsed once with 1 mL lysis buffer and washed 3 times with wash buffer for 5 min tumbling at4°C. Samples were then eluted off the anti-FLAG beads using competitive 500 μg/mL 3xFLAG peptide (Sigma-Aldrich, F4799-4mg) elution in 150 μL lysis buffer by rotation for 1 hour at 4°C. 5% of the elution was used for protein analysis by WB, and 95% was subjected to TRIzol total RNA extraction and library preparation.

#### Library Preparation and Deep Sequencing

5 μg total RNA isolated from FLAG elution samples were treated with Yeast RiboZero Gold (Illumina, MRZY1306) according to the manufacturer’s instructions to remove yeast rRNAs from the samples. From the remaining fragmented RNA in water (10 μL, yield 80-160 ng RNA), 30 ng of elution and mRNA fragment input samples were used for library preparation. Library preparation for deep sequencing was performed using the NextFlex Rapid Directional qRNA-Seq Library Prep Kit (Perkin Elmer, Bioo Scientific, NOVA-5130-01D) according to the manufacturer’s instructions using 7 unique barcodes. In brief, the standard protocol was applied with the following changes: the initial fragmentation step was omitted and PCR amplification was performed using 16 cycles. DNA fragments were purified for Illumina sequencing, subjected to analysis using the High Sensitivity DNA Assay (Agilent) on a Bioanalyzer (Agilent) and all DNA libraries were pooled to a final concentration of 4 nM. Sequencing was performed at the Stanford Functional Genomics Facility (SFGF) at Stanford University, on the Illumina NextSeq 550 instrument, using 2x 75 nt paired-end sequencing and the following library design: AATGATACGGCGACCACCGAGATCTACACTCTTTCCCTACACGACGCTCTTCCGATCTNNNN NNNNT-insert-NNNNNNNNAGATCGGAAGAGCACACGTCTGAACTCCAGTCACBBBBBBBBATCTCGTATGCCGTCTTCTGCTTG, where N is the 2x 8 nt unique molecular index, and B is the 8 nt sample barcode.

#### VELCRO-IP RNA-seq Data Analysis: Read Alignment and Quantification

First, for removal of adaptor sequences, low quality bases, and short reads, we use cutadapt (Martin, 2011) to trim Illumina adaptor sequences and < Q20 bases. Reads < 40 nt were removed. Parameters: cutadapt -m 40 -a AGATCGGAAGAGCACACGTCTGAACTC CAGTCAC -A AGATCGGAAGAGCGTCGTGTAGGGAAAGAGTGTAGATCTCGGTGGTCGCCGTATCATT–nextseq-trim = 20. Next, for UMI extraction, we used umi_tools (Smith et al., 2017) to extract the UMI region (first 8 bases). Parameters: umi_tools extract–bc-pattern = NNNNNNNN–bc-pattern2 = NNNNNNNN. We additionally remove 1 base from 5′ end of the reads, which is the A/T nucleotide overhang from the ligation reaction during library preparation. For splice-aware alignment using STAR (Dobin et al., 2013), we used STAR to align the reads to a reference genome/transcriptome. STAR reference is built using a combination of yeast genome (sacCer3), mouse genome (mm10), mouse rDNA sequence (GenBank: GU372691), and mouse transcript annotations (GENCODE vM18). Only uniquely mapped reads were retained. Parameters: STAR --sjdbOverhang 66 --outFilterMultimapNmax 1 --alignEndsType EndToEnd-alignIntronMax 1000000 --alignMatesGapMax 1000000 --alignIntronMin20-outFilterMismatchNmax 999 --alignSJDBoverhangMin 1 --alignSJoverhangMin 8 --outFilterType BySJout. While the majority of the reads mapped to yeast mRNAsthatwe believe reflect background binding from the initial ribosome-IP (~20 million reads), 1%–3% mapped to mouse mRNAs which corresponds to ~500,000 reads per sample. For deduplication using UMI, we used umi_tools to deduplicate the alignments. Deduplicated alignments are re-aligned using STAR and the same parameters as before. Parameters: umi_tools dedup–paired–buffer-whole-contig. For read quantification, we used bedtools (Quinlan and Hall, 2010) to count alignments over 200 nt sliding windows with step size of 100 nt across mouse genome.

#### VELCRO-IP RNA-seq Data Analysis: Enrichment Analysis

For data matrix and normalization, each cell in the data matrix is the read count, where rows are 200 nt genomic windows and columns are the samples. We discarded rows whose sum of counts across all six mutant and wild-type samples was < 30. We used the TMM (Robinson and Oshlack, 2010) method to calculate normalization factors. Counts divided by normalization factors were used for plotting tracks along the transcript. Tracks are plotted using wiggleplotR (Alasoo et al., 2015). Each genomic window is annotated as 5′ UTR, ORF, or 3′ UTR based on any overlap with any isoform present in the GENCODE vM18 annotation. For statistical significance of enriched windows, we use voom (Law et al., 2014)-limma (Ritchie et al., 2015) to model mean-variance bias and calculate moderated t-statistics and p values for the difference in mutant versus wild-type samples. We noted the heavy tailed histogram of the t-statistics suggesting high proportion of non null windows and used locfdr (Efron, 2010) approach to estimate local false discovery rates. All reported FDR values in the manuscript are locfdr estimates. Locfdr parameters: bre = 150, df = 25, pct = 0, nulltype = 1, type = 0, mlests = (−0.5, 1.0). To test overrepresentation of enriched windows across 5′ UTR-CDS-3′ UTR regions, we performed permutation based chi-square test of independence on the contingency table of regions that the windows overlap versus whether the FDR for enrichment of windows were ≤ 0.05. For Gene Ontology (GO) term enrichment, GO terms and gene mappings were obtained from Bioconductor annotation package org.Mm.eg.db (version 3.6.0). We used topGO (Alexa et al., 2006) to perform enrichment analysis. We choose the combination of Fisher’s exact test and weight01 algorithm for handling local similarities between GO terms. Genes that have at least one window with FDR ≤ 0.05 are used as the positive set. All genes that have at least one window tested are used as the background. For the reported list of GO terms in the manuscript, the following criteria are true: observed/expected ratio > = 2, minimum number of observed genes > = 3, Fisher’s exact test FDR ≤ 0.05, and weight01-conditioned Fisher’s exact test p value ≤ 0.05. FDR for Fisher’s exact test is estimated by permutation of the gene labels of the positive set.

For k-mer enrichment analysis, we first counted the occurrences of all possible substrings (length 4 ≤ k ≤ 8 of the reverse complement sequence of hES9S within each expressed genomic windows tested in differential binding analysis. To test overrepresentation of each k-mer in hES9S-enriched windows, we performed Wilcoxon rank sum test between the vectors of counts of the k-mer across hES9S enriched versus all 5′ UTR windows. False discovery rates were estimated using Benjamini-Hochberg procedure. The k-mers reported as significant k-mers in the manuscript have FDR estimate ≤ 0.05 and location parameter estimate > 0. Significant k-mers with k > = 5 are shown in plots of individual examples of 5′ UTRs.

#### Data Sources

For the multiple sequence alignment (MSA) and conservation analysis of ES9S and surrounding 18S rRNA sequence, the following 18S rRNA sequences were retrieved for eukaryotic species from the NCBI database as data sources and references and aligned by Multiple Alignment using Fast Fourier Transform (MAFFT, MView, EMBL-EBI webtools) with default settings: mouse (*Mus musculus*; GenBank: NR_003278.3), human (*Homo sapiens*; M10098.1), chicken (*Gallus gallus*; AF173612.1), African clawed frog (*Xenopus laevis*; X02995.1), zebrafish (*Danio rerio*; NR_145818.1); juvenile axolotl (*Ambystoma mexicanum*); and yeast (*Saccharomyces cerevisiae*; J01353.1).

### QUANTIFICATION AND STATISTICAL ANALYSIS

In all figures, data was presented as mean, SD or SEM as stated in the figure legends, and *p ≤ 0.05 was considered significant (ns: p > 0.05; *p ≤ 0.05; **p ≤ 0.01; ***p ≤ 0.001; ****p ≤ 0.0001). Blinding and randomization were not used in any of the experiments. Number of independent biological replicates used for the experiments are listed in the figure legends. Tests, two-tailed unpaired Student’s t test if not stated otherwise, and specific p values used are indicated in the figure legends. In all cases, multiple independent experiments were performed on different days to verify the reproducibility of experimental findings. For mouse experiments, embryos from multiple litters were used to avoid litter-specific bias.

## Supplementary Material

1

2

3

## Figures and Tables

**Table:** KEY RESOURCES TABLE

REAGENT or RESOURCE	SOURCE	IDENTIFIER
Antibodies		
Mouse monoclonal anti-FLAG (M2)	Sigma-Aldrich	Cat# F3165; RRID: AB_259529
Mouse monoclonal anti-PGK1	Thermo, Novex	Cat# 459250; RRID: AB_2532235
Mouse monoclonal anti-RPS5/uS7	Abcam	Cat# ab58345; RRID: AB_2180899
Mouse monoclonal anti-RPL10A/uL1 (for yeast)	Santa Cruz	Cat# sc-100827; RRID: AB_2285311
Rabbit monoclonal anti-RPL10A/uL1 (for mouse)	Abcam	Cat# ab174318; RRID: N/A
Rat monoclonal anti-Mouse IgG-HRP (eB144)	Rockland	Cat# 18-8817-31; RRID: AB_2610850
Mouse monoclonal anti-Rabbit IgG-HRP (eB182)	Rockland	Cat# 18-8816-31; RRID: AB_2610847
Sheep Anti-Mouse IgG, HRP Conjugated	GE Healthcare	Cat# NXA931; RRID: AB_772209
Donkey Anti-Rabbit IgG, HRP Conjugated	GE Healthcare	Cat# NA934; RRID: AB_772206
Mouse monoclonal anti-FLAG M2 affinity Gel	Sigma-Aldrich	Cat# A2220; RRID: AB_10063035
Chemicals, peptides, and recombinant proteins		
Cycloheximide	Sigma-Aldrich	Cat# C7698-1G
RNase A	Invitrogen	Cat# AM2271
RNA PureLink columns	Ambion	Cat# 12183018
RNA Clean and Concentrator-5 columns	Zymo Research	Cat# R1016
3xFLAG peptide	Sigma-Aldrich	Cat# F4799-4MG
TURBO DNase	Ambion	Cat# AM2238
SUPERase In RNase Inhibitor	Ambion	Cat# AM2696
RNaseOUT	Thermo Fisher	Cat# 10777019
RNasin Plus RNase inhibitor	Promega	Cat# N261A
TRIzol	Invitrogen	Cat# 15596
AccuPrime Pfx DNA Polymerase	Invitrogen	Cat# 12344024
KOD Xtreme Hot Start DNA Polymerase	EMD Millipore	Cat# 71975
SuperScript III Reverse Transcriptase	Invitrogen	Cat# 18080044
SuperScript IV Reverse Transcriptase	Invitrogen	Cat# 18090010
iScript Supermix	Bio-Rad	Cat# 1708840
SsoAdvanced SYBR Green supermix	Bio-Rad	Cat# 1725270
CFX384 Touch qPCR machine	Bio-Rad	Cat# 1855485
5-Fluoroorotic Acid (5-FOA)	Fisher Scientific	Cat# F10501-5.0
Geneticin	GIBCO	Cat# 11811-031
Amino acid supplements (Complete Supplement Mixture, CSM)	Sunrise Science Products	https://sunrisescience.com/products/growth-media/amino-acid-supplement-mixtures/csm-formulations/
Salmon sperm DNA	Sigma	Cat# D1626-5G
Poly ethylene glycol (PEG) – MW 8000	Millipore Sigma	Cat# 6510-OP
cOmplete Protease Inhibitor Cocktail, EDTA-free	Roche	Cat# 11836145001
cOmplete Mini Protease Inhibitor Cocktail, EDTA-free	Roche	Cat# 11836170001
Streptavidin Sepharose High Performance	GE Healthcare	Cat# 17-5113-01
Avidin Agarose	Thermo, Pierce	Cat# 20219
SDS-PAGE gels	Bio-Rad	Cat# 567-1095, 456-1096
Semi-dry Trans-Blot Turbo system	Bio-Rad	Cat# 170-4273
Clarity Western ECL Substrate	Bio-Rad	Cat# 170-5061
ChemiDoc MP	Bio-Rad	Cat# 17001402
Tissue Lyser (QIAgen TissueLyser II)	QIAGEN	Cat# 85300
Dulbecco’s Modified Eagle’s Medium	GIBCO	Cat# 11965–118
Fetal calf serum	EMD Millipore	Cat# TMS-013-B
Opti-MEM	GIBCO	Cat# 11058-021
Lipofectamine 2000	Invitrogen	Cat# 11668-019
1x PBS	GIBCO	Cat# 14190-250
SYBR Gold	Invitrogen	Cat# S11494
GlycoBlue	Ambion	Cat# LSAM9516
Sucrose	Fisher Scientific	Cat# S5-12
Density Gradient Fraction System	Brandel	Cat# BR-188
Acid-Phenol:Chloroform, pH 4.5	Ambion	Cat# AM9722
Critical commercial assays		
Ribo-Zero Gold rRNA Removal Kit (Yeast)	Illumina	Cat# MRZY1306
NextFlex Rapid Directional qRNA-Seq Library Prep Kit	Perkin Elmer, Bioo Scientific	Cat# NOVA-5130-01D
ProteoExtract Protein Precipitation Kit	EMD Millipore	Cat#539180
Dual-Luciferase Reporter Assay System	Promega	Cat# E1980
GloMax-Multi	Promega	Cat# E7081
MEGAscript T7 Transcription Kit	Ambion	Cat# AM1333
MEGAscript SP6 Transcription Kit	Ambion	Cat# AM1330
MasterPure Yeast RNA Purification Kit	Epicenter	Cat# MPY03100
QIAquick Gel Extraction Kit	QIAgen	Cat# 28706
Monarch Gel Extraction Kit	NEB	Cat# T1020S
NEBuilder HiFi DNA Assembly Master Mix	NEB	Cat# E2621S
QIAquick PCR Purification Kit	QIAgen	Cat# 28106
G-50 Mini Quick Spin Sephadex RNA columns	Roche	Cat# 11814427001
Oligotex mRNA Mini Kit	QIAgen	Cat# 70022
Poly(A) Purist MAG kit	Invitrogen	Cat# AM1922
NEBNext Magnesium RNA Fragmentation Module	NEB	Cat# E6150S
RNA 6000 Pico Chip	Agilent	Cat# 5067-1513
High Sensitivity DNA Assay	Agilent	Cat# 5067-4626
Deposited data		
Raw and analyzed VELCRO-IP RNA-seq data	This paper	GEO: GSE141382
Mouse reference transcriptome mm9 knownGene	UCSC Genome Browser	https://genome.ucsc.edu/cgi-bin/hgTables
Mouse mm9 knownCanonical annotation	UCSC Genome Browser	https://genome.ucsc.edu/cgi-bin/hgTables
Experimental models: cell lines		
C3H/10T1/2 mouse cells	ATCC	Cat# CCL-226
Experimental models: organisms/strains		
Yeast (*S. cerevisiae*) strains used: see Table S2	This paper	N/A
Oligonucleotides		
Oligonucleotides for genome editing, cloning, qRT-PCR analysis, *in vitro* transcription, see Table S3	This paper	N/A
Synthesized oligonucleotides	Twist Bioscience	N/A
Recombinant DNA		
Plasmids used and generated, see Table S1	This paper	N/A
Software and algorithms		
Agilent 2100 Bioanalyzer Expert software	Agilent	https://www.agilent.com/
Cutadapt	Martin, 2011	https://cutadapt.readthedocs.io/en/stable/
FastQC	Babraham Bioinformatics	http://www.bioinformatics.babraham.ac.uk/projects/fastqc/
deepTools	Ramírez et al., 2016	https://github.com/deeptools/deepTools/
UMI-tools	Smith et al., 2017	https://github.com/CGATOxford/UMI-tools
Locfdr	Efron, 2010	https://cran.r-project.org/web/packages/locfdr/index.html
STAR RNA-Seq aligner	Dobin et al., 2013	https://github.com/alexdobin/STAR
bedtools	Quinlan and Hall, 2010	https://bedtools.readthedocs.io/en/latest/
Samtools	Li et al., 2009	http://samtools.sourceforge.net/
TMM	Robinson and Oshlack, 2010	N/A
wiggleplotR	Alasoo et al., 2015	https://bioconductor.org/packages/release/bioc/html/wiggleplotr.html
voom	Law et al., 2014	N/A
limma	Ritchie et al., 2015	https://bioconductor.org/packages/release/bioc/html/limma.html
topGO	Alexa et al., 2006	https://bioconductor.org/packages/release/bioc/html/topGO.html
MAFFT, MView	EMBL-EBI webtools	https://www.ebi.ac.uk/Tools/msa/mafft/
Vienna RNAfold	RNAfold WebServer	http://rna.tbi.univie.ac.at
VARNA	RNA structure visualization	http://varna.lri.fr
R	R Foundation	https://www.r-project.org/
ImageJ	NIH	https://imagej.nih.gov/ij/
Prism	GraphPad Software Inc.	Version 8.0

## Highlights

- Engineering of yeast ribosomes with species-specific rRNA ESs
- Humanized ES9S yeast ribosomes directly recruit select mRNAs
- Genome-wide identification of mRNA regions that specifically bind ribosomes via ES9S
- ES9S-interacting mammalian 5′ UTRs mediate cap-independent translation
