# Supplementary material for: VELCRO-IP RNA-seq reveals ribosome expansion segment function in translation genome-wide
Source: Cell Rep. Author manuscript; Available in PMC 2021 Jul 9. (PMC8270675; doi:10.1016/j.celrep.2020.108629)
Supplement: 1 [file NIHMS1664954-supplement-1.pdf]

Cell Reports, Volume *34*

Supplemental Information

VELCRO-IP RNA-seq reveals ribosome expansion segment function in translation genome-wide

Kathrin Leppek, Gun Woo Byeon, Kotaro Fujii, and Maria Barna

SUPPLEMENTAL INFORMATION

VELCRO-IP RNA-seq reveals ribosome expansion segment function in translation genome-wide

Kathrin Leppek[1,2,3], Gun Woo Byeon[1,2,3], Kotaro Fujii[1,2], Maria Barna[1,2,4,*]

[1] Department of Developmental Biology, Stanford University, Stanford, California 94305, USA
[2] Department of Genetics, Stanford University, Stanford, California 94305, USA
[3] These authors contributed equally
[4] Lead Contact
[*] Correspondence: mbarna@stanford.edu

This document includes:

Figures S1 to S6
Tables S1 to S3

Other supplementary material for this manuscript includes the following:

Table S4:	mRNA regions enriched by VELCRO-IP RNA-seq
Table S5:	GO term analysis for all genes enriched with hES9S

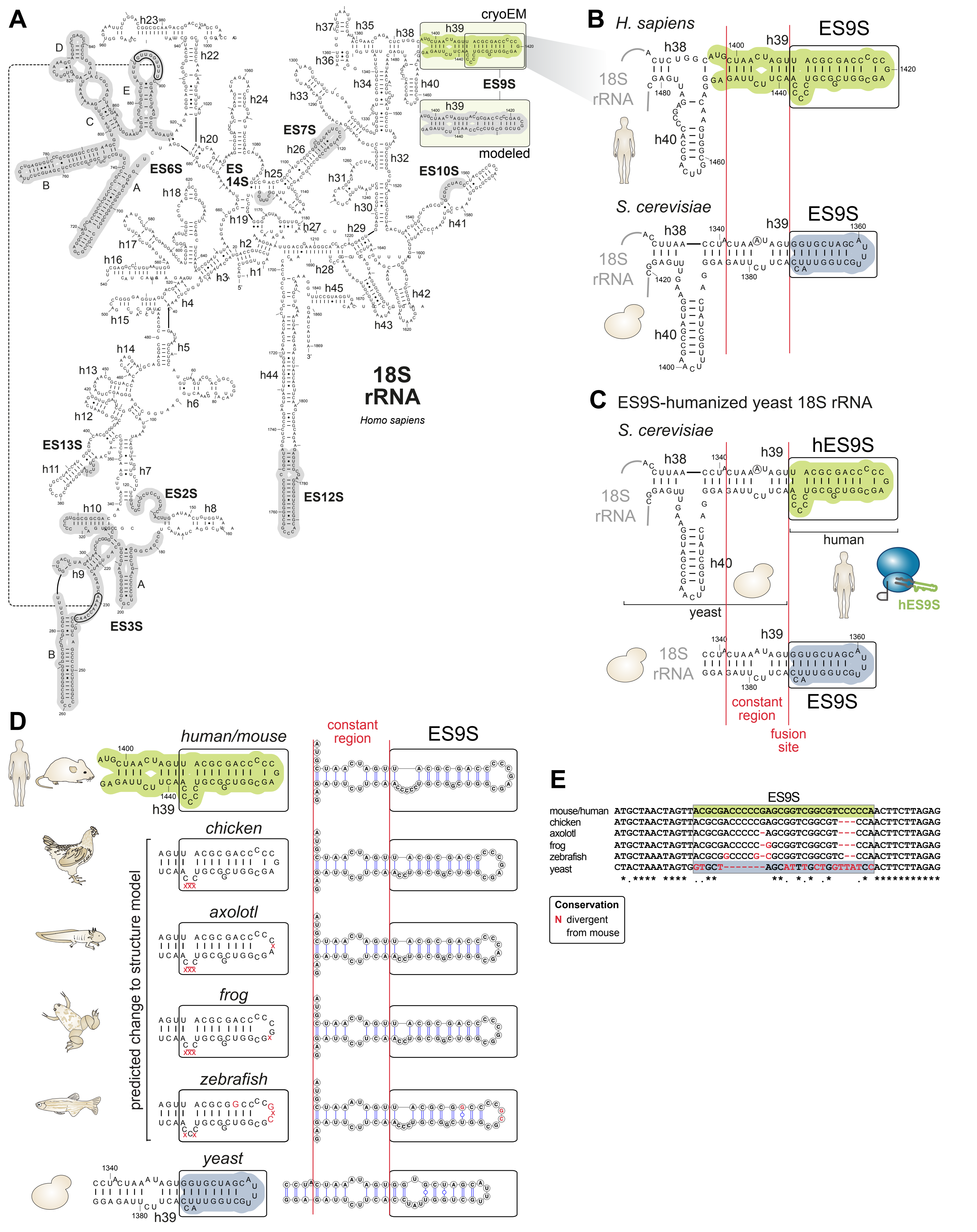
A
18S rRNA
Homo sapiens
cryoEM
modeled
h39
ES9S
ES6S
ES7S
ES10S
ES 14S
ES13S
ES2S
ES3S
ES12S
h1 h2 h3 h4 h5 h6 h7 h8 h9 h10 h11 h12 h13 h14 h15 h16 h17 h18 h19 h20 h22 h23 h24 h25 h26 h27 h28 h29 h30 h31 h32 h33 h34 h35 h36 h37 h38 h39 h40 h41 h42 h43 h44 h45
B
H. sapiens
S. cerevisiae
18S rRNA
h38
h39
h40
ES9S
C
ES9S-humanized yeast 18S rRNA
S. cerevisiae
hES9S
human
yeast
constant region
fusion site
D
human/mouse
chicken
axolotl
frog
zebrafish
yeast
predicted change to structure model
constant region
ES9S
E
ES9S
mouse/human ATGCTAACTAGTTACGCGACCCCCGAGCGGTCGGCGTCCCCCAACTTCTTAGAG
chicken ATGCTAACTAGTTACGCGACCCCCGAGCGGTCGGCGT---CCAACTTCTTAGAG
axolotl ATGCTAACTAGTTACGCGACCCCC-AGCGGTCGGCGT---CCAACTTCTTAGAG
frog ATGCTAACTAGTTACGCGACCCCC-GGCGGTCGGCGT---CCAACTTCTTAGAG
zebrafish ATGCTAAATAGTTACGCGGCCCCG-CGCGGTCGGCGTC--CCAACTTCTTAGAG
yeast CTACTAAATAGTGGTGCT-------AGCATTTGCTGGTTATCCACTTCTTAGAG
Conservation
N divergent from mouse

Figure S1. Confirmation of interspecies sequence variation of ES9S rRNA region. Related to Figure 1.

(**A**) Secondary structure model of the human (*H. sapiens*) 18S rRNA adapted from (Anger et al., 2013). rRNA expansion segment regions are highlighted in grey. Nucleotide positions, helices and ESs are numbered. The boxed region shows the ES9S structure based on either our cryo-EM data (green; (Leppek et al., 2020)) or based on a previous model (grey; (Anger et al., 2013)).
(**B**) Secondary structure models of the human and baker's yeast (*S. cerevisiae*) 18S rRNA region containing ES9S, highlighted in green and blue, respectively. The structure of the distal human ES9S (boxed region in A and B) was revised based on cryo-EM data (Leppek et al., 2020).
(**C**) Secondary structure model of the engineered yeast 18S rRNA after exchange of the yeast ES9S with the human one (hES9S, green). Constant region (h39) and ES9S-fusion site selected for engineering chimeric 18S rRNA are indicated in red.
(**D**) Predicted structural changes in the ES9S region of 18S rRNA across different species. Sequence changes and their predicted effects on the ES9S structure are indicated in red. Human/mouse ES9S (identical sequence) is the reference for the comparison. The variable sequences across the species are obtained by RT-PCR from total RNA extracts of the different species (E11.5, stage E11.5 FVB mouse embryo; chicken, *Gallus gallus*; axolotl, *Ambystoma mexicanum*; frog, X. l., *Xenopus laevis*; zebrafish, *Danio rerio*; yeast, S. c., *Saccharomyces cerevisiae*) using primers specific for the 18S rRNA region containing ES9S in the center (see **Figure 1A-C**, partially reproduced from **Figure 1A**). Secondary structures of ES9S of different species were modeled using Vienna RNAfold (http://rna.tbi.univie.ac.at) and visualized using VARNA (http://varna.lri.fr) with default settings.
(**E**) Multiple sequence alignment of RT-PCR-confirmed ES9S sequences from the different species used for the structure models in (D). Partially reproduced from **Figure 1C**.

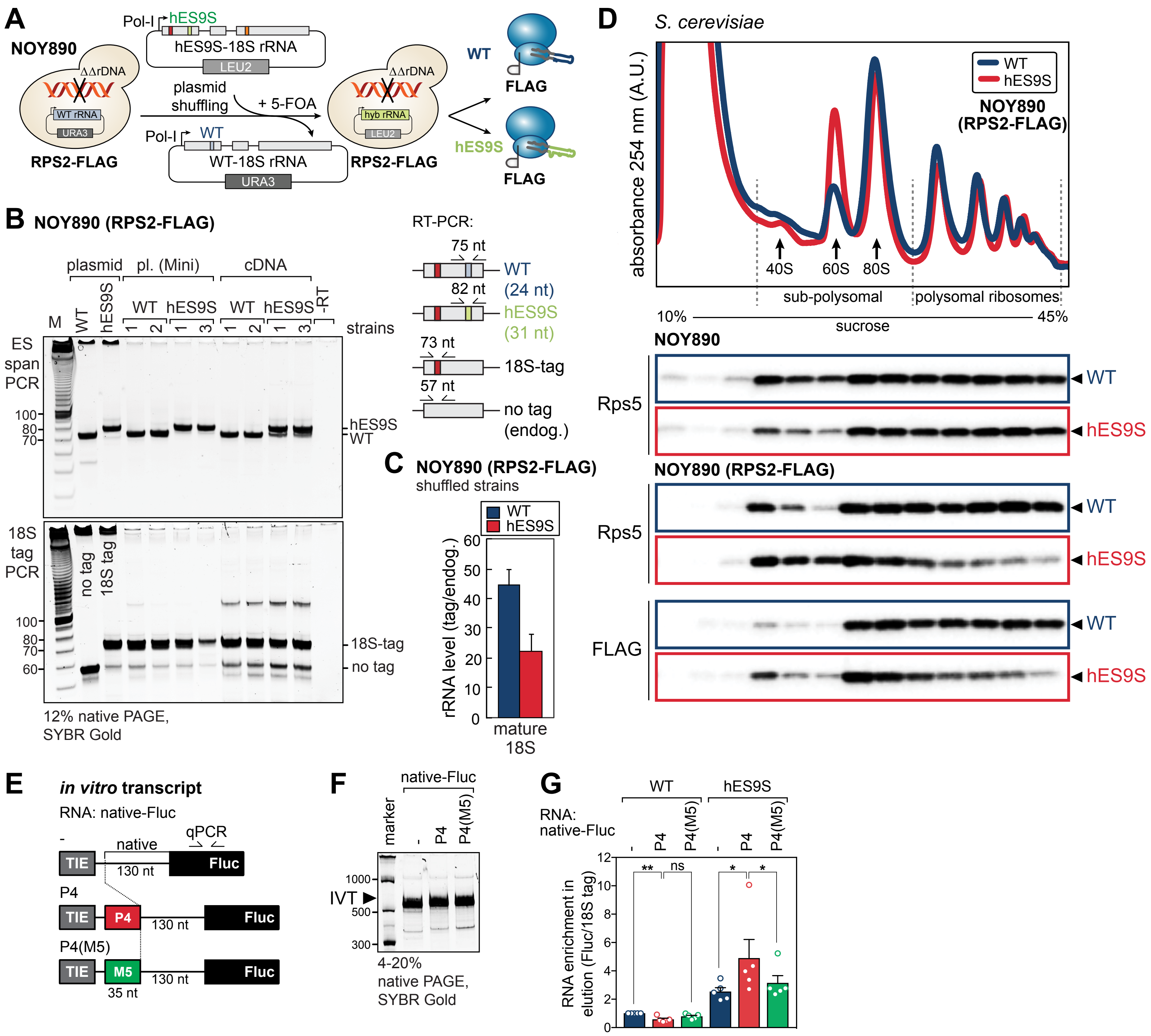
A
NOY890
ΔΔrDNA
WT rRNA
URA3
RPS2-FLAG
Pol-I hES9S
hES9S-18S rRNA
LEU2
plasmid shuffling
+ 5-FOA
Pol-I WT
WT-18S rRNA
URA3
hyb rRNA
LEU2
RPS2-FLAG
WT
FLAG
hES9S
FLAG
B NOY890 (RPS2-FLAG)
plasmid
pl. (Mini)
cDNA
M
WT
hES9S
WT hES9S
WT hES9S
-RT
1 2 1 3 1 2 1 3
strains
ES span PCR
100
80
70
hES9S
WT
18S tag PCR
no tag
18S tag
100
80
70
60
18S-tag
no tag
12% native PAGE, SYBR Gold
RT-PCR:
75 nt
WT (24 nt)
82 nt
hES9S (31 nt)
73 nt
18S-tag
57 nt
no tag (endog.)
C NOY890 (RPS2-FLAG)
shuffled strains
WT
hES9S
rRNA level (tag/endog.)
0
10
20
30
40
50
60
mature 18S
D S. cerevisiae
absorbance 254 nm (A.U.)
WT
hES9S
NOY890 (RPS2-FLAG)
40S
60S
80S
sub-polysomal
polysomal ribosomes
10%
sucrose
45%
NOY890
Rps5
WT
hES9S
NOY890 (RPS2-FLAG)
Rps5
WT
hES9S
FLAG
WT
hES9S
E in vitro transcript
RNA: native-Fluc
-
TIE
native
130 nt
qPCR
Fluc
P4
TIE
P4
130 nt
Fluc
P4(M5)
TIE
M5
130 nt
Fluc
35 nt
F native-Fluc
marker
-
P4
P4(M5)
1000
IVT
500
300
4-20% native PAGE, SYBR Gold
G
RNA: native-Fluc
WT
hES9S
-
P4
P4(M5)
-
P4
P4(M5)
RNA enrichment in elution (Fluc/18S tag)
0
2
4
6
8
10
12
**
ns
*
*

Figure S2. Plasmid shuffling and yeast strain characterization; and VELCRO-IP qRT-PCR serves as a proof-of-principle to identify novel hES9S-interacting 5' UTRs. Related to Figure 1, 2, 3.

(**A**) A yeast strain containing the plasmid-encoded chimeric 18S rRNA is generated by plasmid shuffling. Schematic of the plasmid shuffling approach to generate yeast strains (NOY890, RPS2-FLAG) that contain a homozygous knock-out of the rDNA locus (NOY890), resulting in rRNA transcription exclusively from the plasmids. All rDNA plasmids contain unique 18S and 25S rRNA sequence tags. 5-FOA-based selection of transformed yeast cells allows for isolation of clones that retain a transformed *LEU2*-plasmid (pNOY373) and lost the original *URA3*-plasmid (pNOY373). Successful plasmid exchange from *URA3* (WT) to *LEU2* (tagged WT or hES9S)-plasmids in isolates is achieved by growth on SD-*LEU2*, and SD+5-FOA but not on SD-*LEU/URA*.

(**B**) RT-PCR analysis using ES9S-specific primers that span ES9S allow analysis of expression of WT or hES9S 18S rRNA since there is a 7 nt difference in the length of the PCR products between WT and hES9S (ES span PCR). Similarly, the presence of the 18S tag can be distinguished from WT rRNA (18S tag PCR). Total RNA for cDNA synthesis or plasmid DNA was extracted from clones and used for RT-PCR. Plasmid-derived PCR products serve as controls. PCR products were resolved by 12% native PAGE and stained with SYBR Gold. Two independent isolates of tagged-WT and tagged-hES9S strains (NOY890/RPS2-FLAG background) used in this study are presented. RT-PCR specific for the 18S rRNA tag confirms the presence of the tag in transformed plasmid-derived mature 18S rRNA. A 10 bp DNA ladder (Invitrogen) was loaded as reference.

(**C**) Yeast strain characterization after plasmid shuffling and isolation of clones. qRT-PCR analysis with specific primers for rRNA tags and endogenous rRNAs is used to quantify tag/endogenous rRNA levels (i.e. the substitution rates of WT with tagged-WT or tagged-hES9S ribosomes present in isolated strains). For NOY890/RPS2-FLAG strains, the qRT-PCR analysis determined that only one endogenous plasmid-derived WT ribosomes still remained per every 44 tagged WT or every 22 tagged hES9S ribosomes.

(**D**) Sucrose gradient fractionation analysis of yeast lysates derived from WT and hES9S-stains in the background of NOY890 and NOY890/RPS2-FLAG, containing scarless C-terminal Rps2-FLAG (Jan et al., 2014), on 10-45% sucrose gradients (n = 3). Compared to WT rRNA-containing cells, humanized ribosome-containing cells show a slight growth defect. Polysome traces demonstrate proper ribosomal assembly. Incorporation of the FLAG tag into polysomes demonstrates its non-perturbative nature.

(**E**) Schematic of *in vitro* transcripts used for the proof-of-principle experiment of the VELCRO-IP qRT-PCR. Reproduced from **Figure 3B**.

(**F**) For qualitative analysis of the integrity of *in vitro* transcripts, RNAs were subjected to 4-20% polyacrylamide/TBE/native PAGE and visualized by SYBR Gold staining.

(**G**) Analysis of total RNA in the 3xFLAG peptide elution by qRT-PCR using same volumes of RNA per sample for the RT. Normalization of Ct values for Fluc to the 18S rRNA tag internally controls for ribosome-IP efficiency per sample. The native/WT sample was used to normalize for fold enrichment of RNA binding (set to 1). The same data as in **Figure 3D** is plotted differently. Average RNA fold enrichment, SEM, n = 5; ns, not significant.

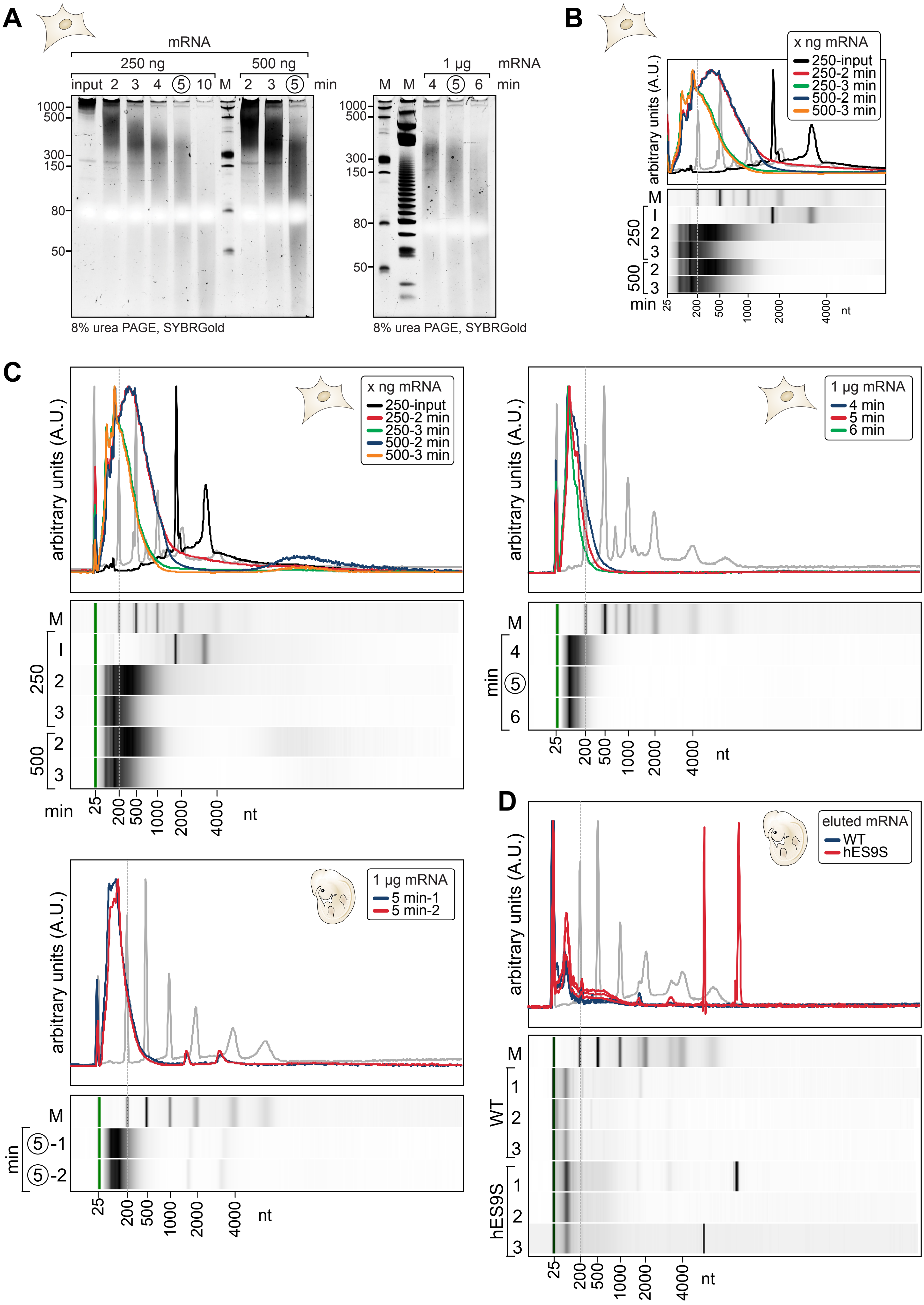
A
mRNA
250 ng
500 ng
input 2 3 4 5 10 M 2 3 5 min
1000
500
300
150
80
50
8% urea PAGE, SYBRGold
1 µg mRNA
M M 4 5 6 min
1000
500
300
150
80
50
8% urea PAGE, SYBRGold
B
x ng mRNA
250-input
250-2 min
250-3 min
500-2 min
500-3 min
arbitrary units (A.U.)
M
I
250
500
min
25 200 500 1000 2000 4000 nt
C
x ng mRNA
250-input
250-2 min
250-3 min
500-2 min
500-3 min
arbitrary units (A.U.)
M
I
250
500
min
25 200 500 1000 2000 4000 nt
1 µg mRNA
4 min
5 min
6 min
arbitrary units (A.U.)
M
4
5
6
min
25 200 500 1000 2000 4000 nt
1 µg mRNA
5 min-1
5 min-2
arbitrary units (A.U.)
M
5-1
5-2
min
25 200 500 1000 2000 4000 nt
D
eluted mRNA
WT
hES9S
arbitrary units (A.U.)
M
WT
hES9S
1
2
3
25 200 500 1000 2000 4000 nt

Figure S3. Controlled mRNA fragmentation. Related to Figure 3.

(**A**) Fragmented mouse mRNA from C3H/10T1/2 cells in different amounts (250 ng, 500 ng, and 1 μg) and timepoints of fragmentation, analyzed by 8% denaturing urea PAGE and visualized by SYBR Gold. The left-most lane shows the 250 ng mRNA input without fragmentation for reference. Ladders: Low Range ssRNA Ladder (NEB); 20 bp Bayou DNA Ladder (Bayou Biolabs).

(**B**) Fragmented mouse mRNA from C3H/10T1/2 cells in different amounts (250 and 500 ng) and timepoints of fragmentation (2 and 3 min) and the 250 ng mRNA input, analyzed on a mRNA Pico Chip (Agilent) on a Bioanalyzer (Agilent). Zoomed-in view of the Bioanalyzer quantification (top) and virtual gel images (bottom) is shown. Grey line plots the marker (lane M in virtual gel images) for reference. See also (**C**).

(**C**) Optimization of mouse mRNA fragmentation from C3H/10T1/2 cells and stage E11.5 mouse embryos. Full views of the Bioanalyzer (Agilent) analyses shown in **Figures S3B, 3F and 3G.** Grey lines plot the markers (lane M in virtual gel images) for reference.

(**D**) Full view of the Bioanalyzer (Agilent) quantification and virtual gel images in **Figure 4B** is shown for the eluted and yeast rRNA-depleted mouse embryo RNA from three independent replicates of WT and hES9S VELCRO-IP experiments. Grey lines plot the markers (lane M in virtual gel images) for reference.

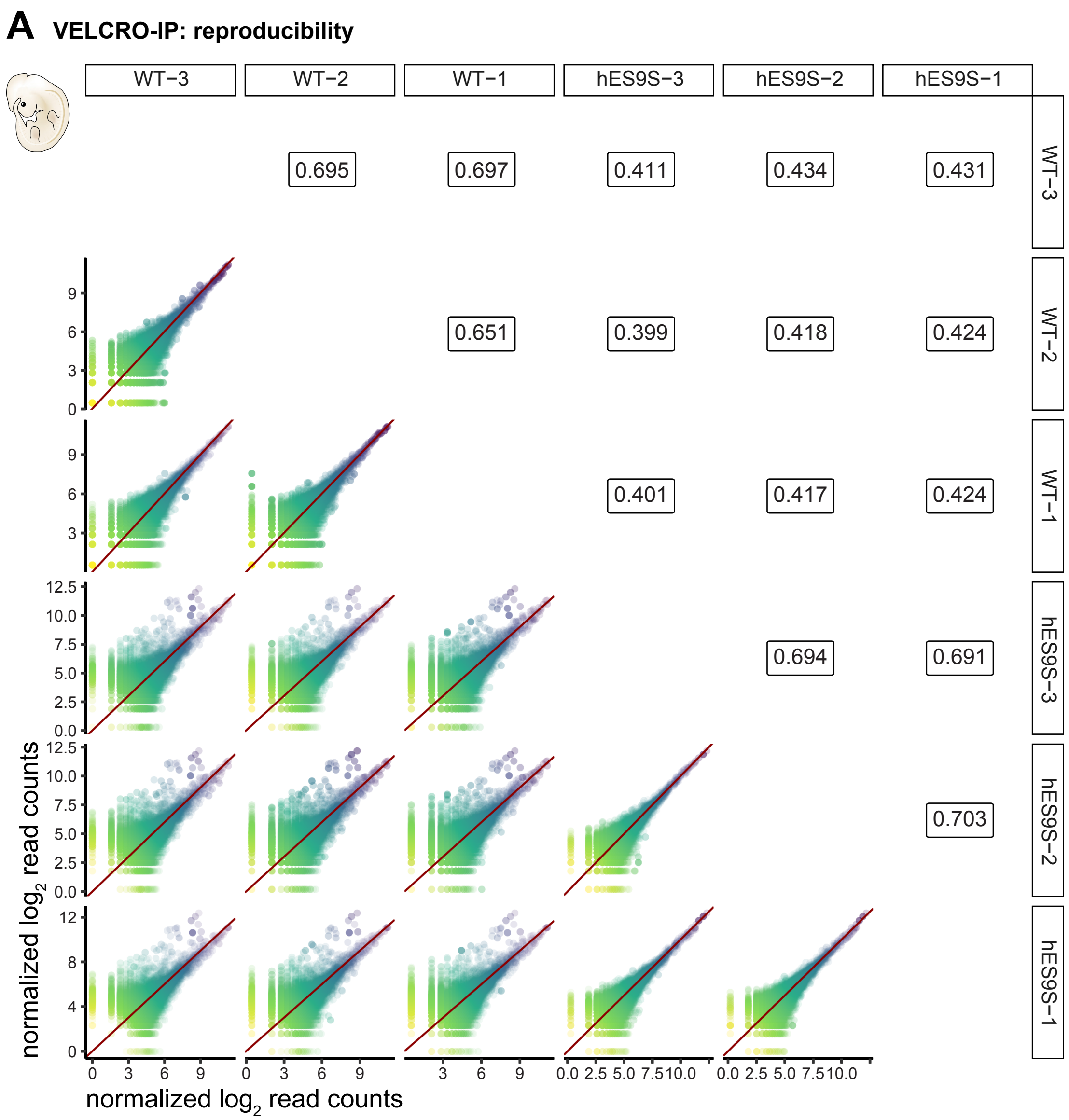
A
VELCRO-IP: reproducibility
WT−3
WT−2
WT−1
hES9S−3
hES9S−2
hES9S−1
0.695
0.697
0.411
0.434
0.431
0.651
0.399
0.418
0.424
0.401
0.417
0.424
0.694
0.691
0.703
normalized log2 read counts
normalized log2 read counts

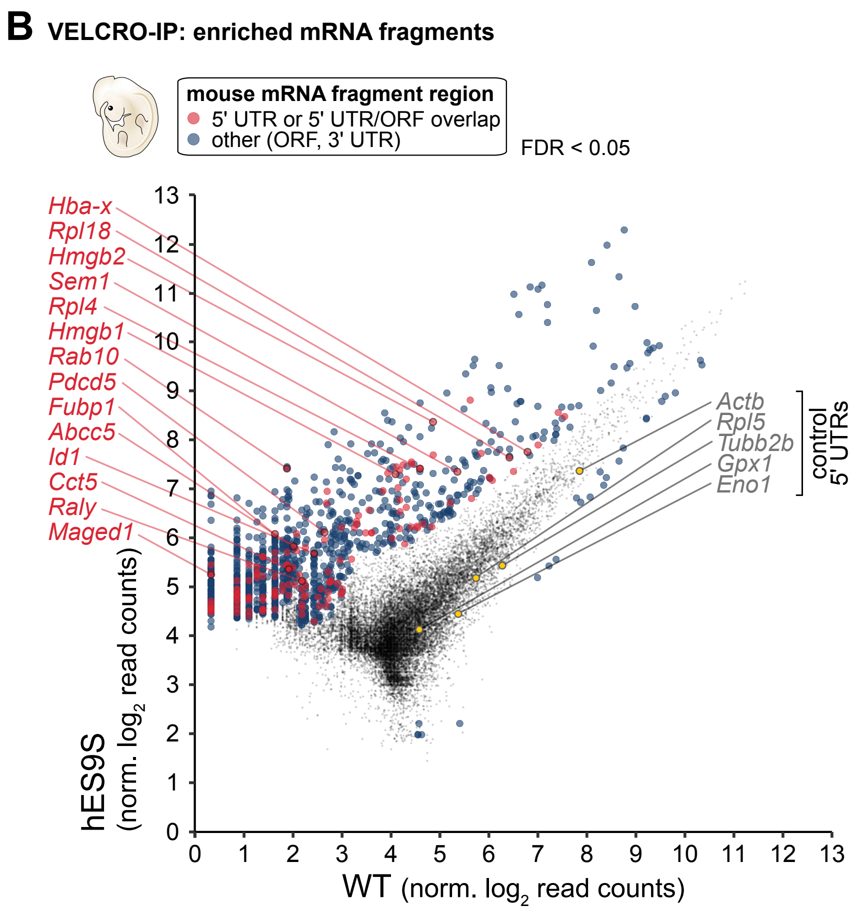
B
VELCRO-IP: enriched mRNA fragments
mouse mRNA fragment region
5' UTR or 5' UTR/ORF overlap
other (ORF, 3' UTR)
FDR < 0.05
Hba-x
Rpl18
Hmgb2
Sem1
Rpl4
Hmgb1
Rab10
Pdcd5
Fubp1
Abcc5
Id1
Cct5
Raly
Maged1
Actb
Rpl5
Tubb2b
Gpx1
Eno1
control 5' UTRs
hES9S (norm. log2 read counts)
WT (norm. log2 read counts)

Figure S4. Reproducibility of VELCRO-IP RNA-seq and identification of hES9S-interacting 5' UTRs. Related to Figure 4.

(**A**) A matrix comparing every possible pair of individual VELCRO-IP RNA-seq samples (three replicate samples per condition, hES9S and WT). Lower triangle: scatter plots of normalized log read counts, colored by expression level. Upper triangle: Pearson correlation coefficient.

(**B**) RNA-seq results of independent replicates (n = 3) for each WT and hES9S samples. Normalized log read counts are presented for WT and hES9S-enriched mRNA fragments. Fragments less than FDR < 0.05 are colored according to the region in the mRNA. Fragments mapping to 5' UTR and overlapping 5' UTR/ORF (red) are highlighted compared to other regions (ORF and 3' UTR, blue). We label mouse genes for which we identified enriched fragments in the 5' UTR and/or 5' region of the ORF and for whose 5' UTRs we performed validation experiments. Five control 5' UTRs are highlighted in yellow that are equally bound to both WT and hES9S 40S subunits and served as negative controls. Corresponds to **Figure 4E**. See also **Table S4**.

A **GO term (biological process) for enriched ORF**

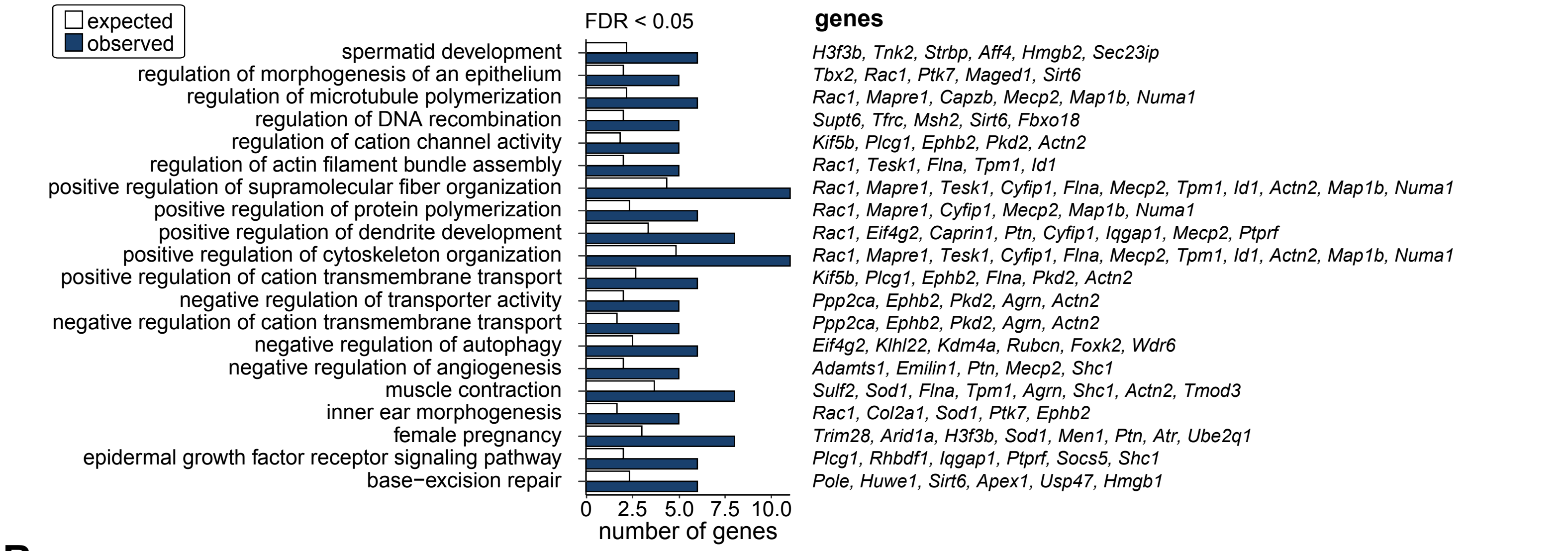

B **GO term (biological process) for enriched 3' UTRs**

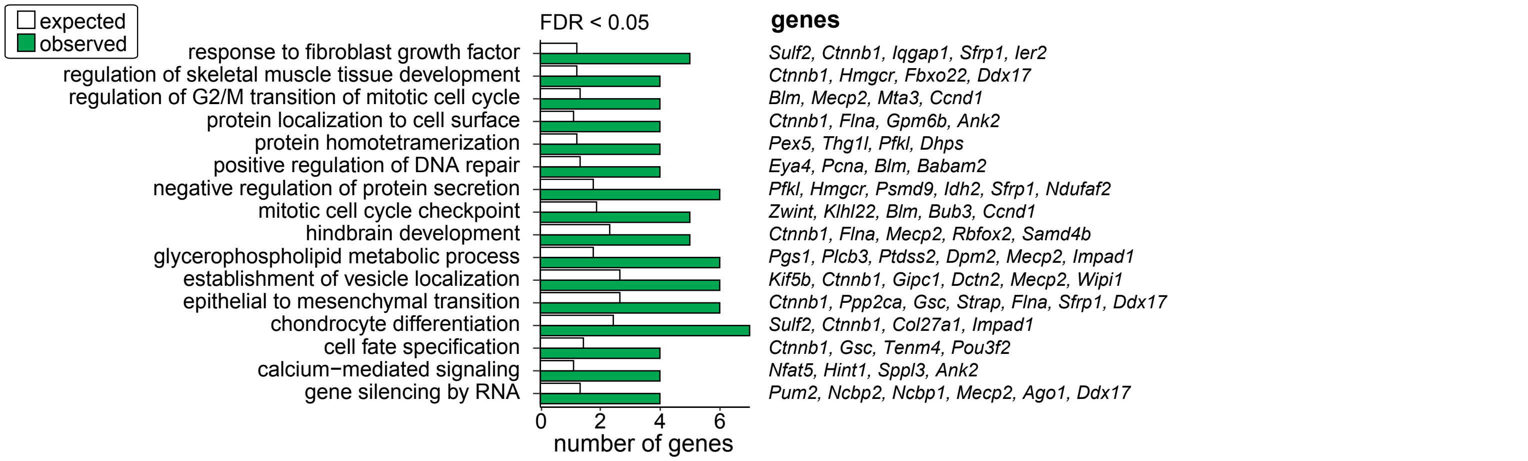

C **GO term (biological process) for enriched mRNA (all regions)**

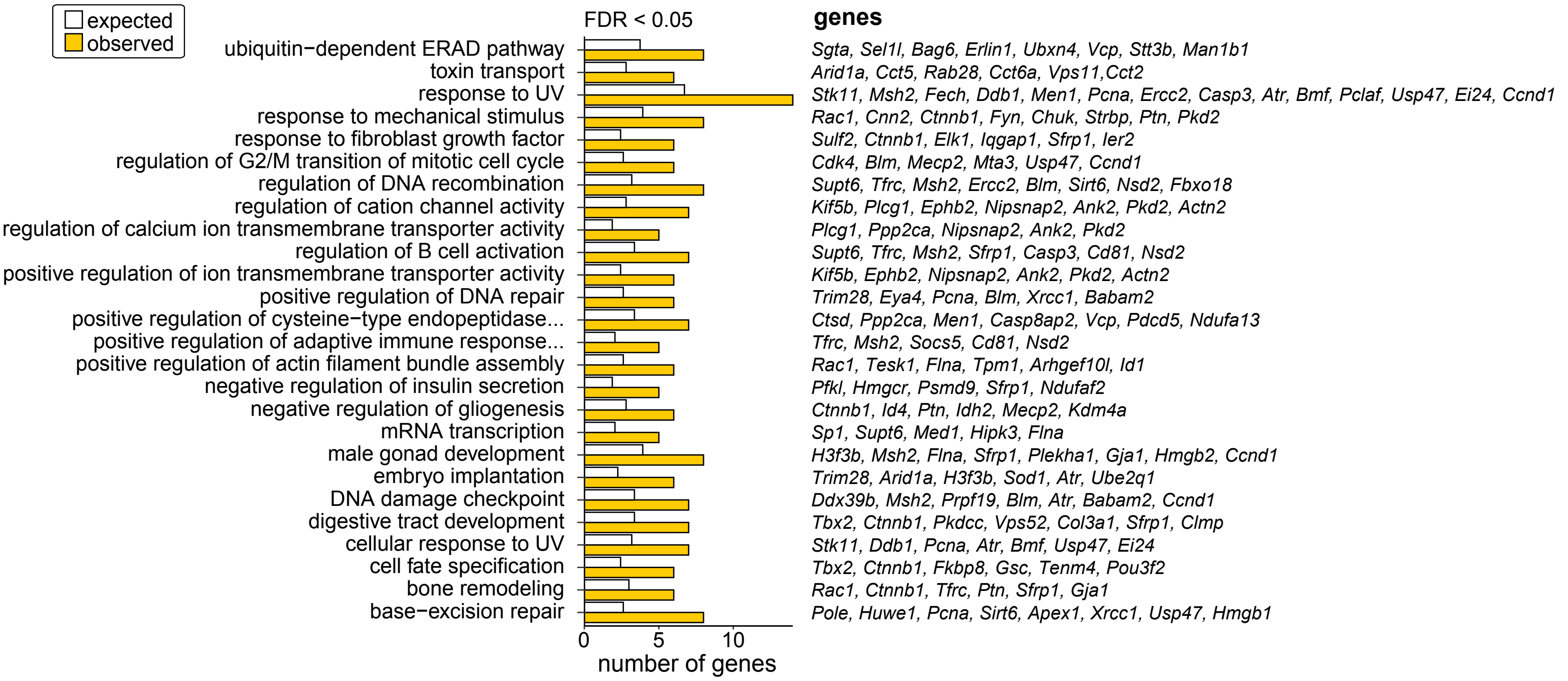

Figure S5. GO-terms of hES9S-interacting mRNA regions. Related to Figure 4.

(**A**) GO term analysis as in **Figure 4H** for biological process of ORF regions (FDR < 0.05, n = 3) enriched by hES9S. Displayed are the expected and observed frequency of genes for the significant terms (FDR < 0.05, expressed mRNA regions were used as the background; see methods for details of the thresholds used). Also see **Table S5**.

(**B**) GO term analysis as in (A) for biological process of 3' UTR regions (FDR < 0.05, n = 3) enriched by hES9S.

(**C**) GO term analysis as in (A) for biological process of the full mRNA (any region) (FDR < 0.05, n = 3) enriched by hES9S.

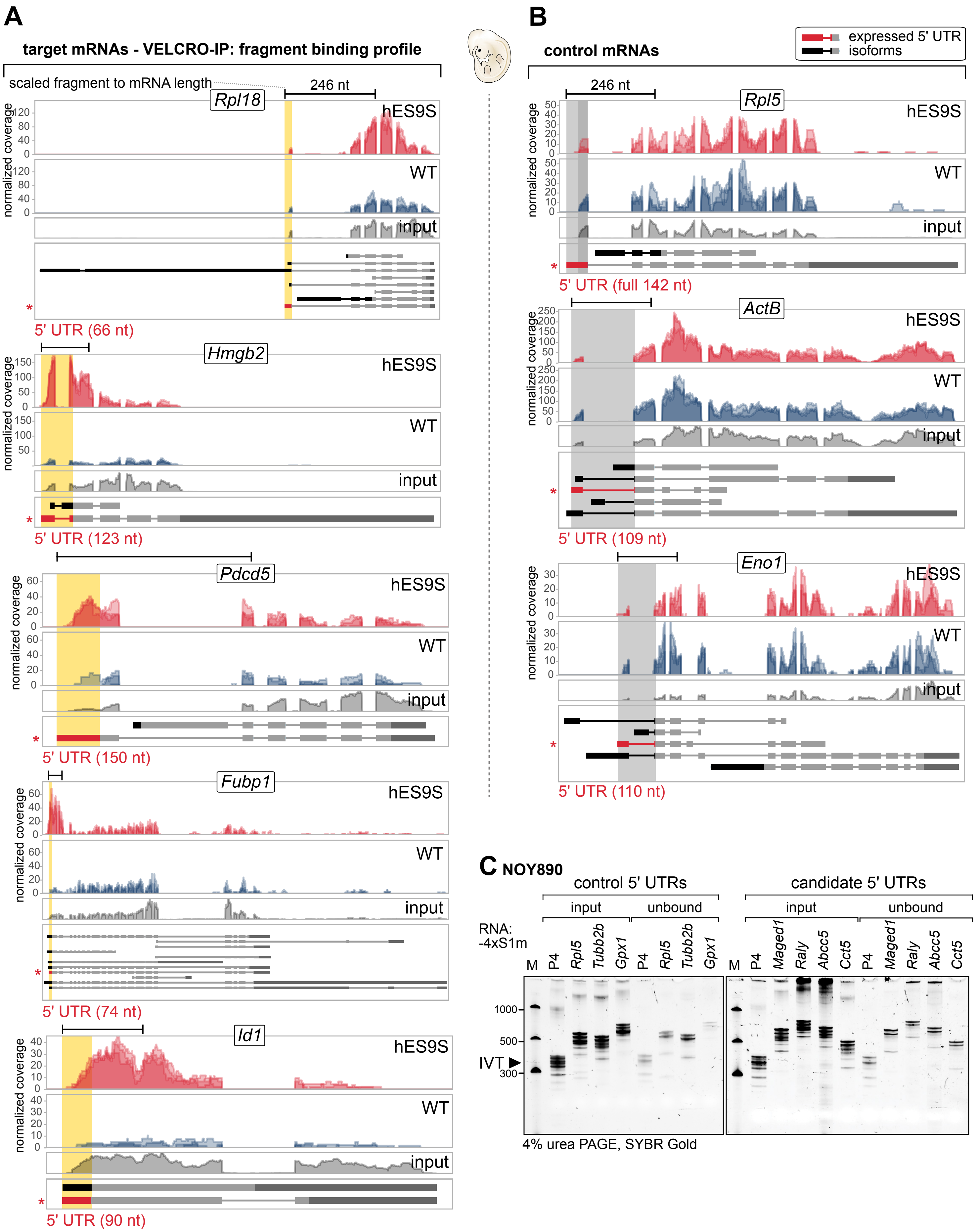
A
target mRNAs - VELCRO-IP: fragment binding profile
scaled fragment to mRNA length
246 nt
Rpl18
Hmgb2
Pdcd5
Fubp1
Id1
hES9S
WT
input
normalized coverage
5' UTR (66 nt)
5' UTR (123 nt)
5' UTR (150 nt)
5' UTR (74 nt)
5' UTR (90 nt)
B
control mRNAs
expressed 5' UTR
isoforms
246 nt
Rpl5
ActB
Eno1
5' UTR (full 142 nt)
5' UTR (109 nt)
5' UTR (110 nt)
C NOY890
control 5' UTRs
candidate 5' UTRs
input
unbound
RNA: -4xS1m
M
P4
Rpl5
Tubb2b
Gpx1
Maged1
Raly
Abcc5
Cct5
1000
500
IVT
300
4% urea PAGE, SYBR Gold

Figure S6. VELCRO-IP mRNA binding pattern and validation of hES9S-interacting 5' UTRs. Related to Figure 5, 6.

(**A**) mRNA binding profile as coverage plots for candidate hES9S-target genes whose 5' UTR-overlapping windows are significantly enriched in the hES9S over WT samples (FDR < 0.05, n = 3). The other five out of the total tested 14 genes not shown in **Figure 5C, D** are shown here. Normalized per base coverage of individual biological replicate libraries for WT (blue) and hES9S (red) samples is plotted (above). All mRNA isoforms annotated in ENSEMBL are displayed below. Exon lengths are to scale while intron lengths are pseudo-scaled. The read coverage of the input mRNA fragments (grey) are also plotted for reference. 5' UTR regions for the most likely expressed mRNA isoform in embryos is highlighted in red and the corresponding regions in the tracks is shaded in yellow. The 5' UTR region picked for further experimental validation corresponds to the asterisk-marked isoform. The mRNA fragment length for each gene is scaled according to the mRNA length for the individual genes presented. See also **Figure 5C**.

(**B**) The same analysis as in (A) was performed for the other three of total five control 5' UTRs where no enrichment of hES9S interaction over WT was found. 5' UTR regions for the most likely expressed mRNA isoform in embryos is highlighted in red and the corresponding regions in the tracks are shaded in gray. Corresponds to **Figure 5D**.

(**C**) A 4xS1m pulldown experiment with the focus on the comparison of full-length control and candidate hES9S-interacting 5' UTRs for their ability to bind to tagged-WT and tagged-humanized 40S subunits was performed. *In vitro* transcribed RNAs fused to 4xS1m aptamers were coupled to SA-sepharose beads for 4xS1m pulldown using WT and hES9S ribosome expressing yeast strains to generate cellular extracts as input. Coupled beads were incubated with cell extracts, washed and eluted using RNase A to release RNA-bound proteins. Input and unbound samples were taken before and after incubation of RNAs with beads. To monitor coupling efficiency, 10% of the input and unbound RNA fraction of each sample was resolved by 4% denaturing polyacrylamide/TBE/urea PAGE and visualized by SYBR Gold. Representative of n = 3 is shown. Low Range ssRNA Ladder (NEB) was loaded for reference. Corresponds to **Figure 6B**.

SUPPLEMENTAL TABLES

Table S1: Plasmids used in this study. Related to STAR Methods.

All plasmids used for *in vitro* transcription and mammalian transient transfection or yeast transformation are listed in the table.

Table S1. List of plasmids		
**Plasmid**	**Notes**	**Reference**
***In vitro* transcription constructs**		
pSP73	SP6 promoter, kindly provided by G. Stoecklin	Promega
pSP73-4xS1m	p2880, kindly provided by G. Stoecklin	(Leppek and Stoecklin, 2014)
pSP73-4xS1m(MCS)		(Leppek et al., 2020)
pSP73-a9(P4)-4xS1m(MCS)		(Leppek et al., 2020)
pSP73-Rpl5-4xS1m(MCS)		This study
pSP73-Tubb2b-4xS1m(MCS)		This study
pSP73-Gpx1-4xS1m(MCS)		This study
pSP73-Maged1-4xS1m(MCS)		This study
pSP73-Raly-4xS1m(MCS)		This study
pSP73-Abcc5-4xS1m(MCS)		This study
pSP73-Cct5-4xS1m(MCS)		This study
Mammalian cells		
**Expression constructs**		
pRF	SV40 promoter, kindly provided by D. Ruggero	
pRF-HCV IRES	kindly provided by D. Ruggero	
pRF-EMCV IRES	kindly provided by D. Ruggero	
pRF-a9-IRES FL		(Xue et al., 2015)
pRF-a9-P4-native		(Leppek et al., 2020)
pGL3-FLB-stop-TIE-native		(Leppek et al., 2020)
pGL3-FLB-stop-TIE-P4-native		(Leppek et al., 2020)
pGL3-FLB-stop-TIE-P4(M5)-native		(Leppek et al., 2020)
pRF-Abcc5	full-length 5’ UTR, 199 nt	This study
pRF-Raly	full-length 5’ UTR, 289 nt	This study
pRF-Cct5	full-length 5’ UTR, 99 nt	This study
pRF-Maged1	184 nt most 3’ of full-length 5’ UTR, 184 nt	This study
pRF-Rpl18	full-length 5’ UTR, 66 nt	This study
pRF-Hmgb2	full-length 5’ UTR, 123 nt	This study
pRF-Pdcd5	full-length 5’ UTR, 150 nt	This study
pRF-Fubp1	full-length 5’ UTR, 74 nt	This study
pRF-Id1	full-length 5’ UTR, 90 nt	This study
pRF-Hba-x	full-length 5’ UTR, 264 nt	This study
pRF-Rab10	200 nt most 3’ of full-length 5’ UTR, 200 nt	This study
pRF-Sem1	full-length 5’ UTR, 104 nt	This study

pRF-Hmgb1	full-length 5' UTR, 155 nt	This study
pRF-Rpl4	full-length 5' UTR, 56 nt	This study
pRF-Rpl5	full-length 5' UTR, 142 nt	This study
pRF-ActB	full-length 5' UTR, 109 nt	This study
pRF-Tubb2b	full-length 5' UTR, 121 nt	This study
pRF-Eno1	full-length 5' UTR, 110 nt	This study
pRF-Gpx1	full-length 5' UTR, 238 nt	This study
Yeast		
**rDNA constructs**		
pNOY373-18S25Stag	*LEU2, 2μ, Pol1-rDNA- tagged rRNA*	(Leppek et al., 2020)
pNOY373-18S25Stag-hES9S	*LEU2, 2μ, Pol1-rDNA- tagged rRNA-hES9S*	(Leppek et al., 2020)

Table S2: Yeast strains used in this study. Related to STAR Methods.

All yeast strains used and/or generated for this study are listed in the table.

Table S2. List of yeast strains

Strain	Genotype and Notes	Reference
KAY488 (NOY890)	*MATA ura3-1 leu2-3,112 his3-11 trp1-1 ade2-1 can1-100 rdnaΔΔ::HIS3 carrying pRDN-hyg::URA3*	(Nemoto et al., 2010)
NOY890 WT rRNA	*MATA ura3-1 leu2-3,112 his3-11 trp1-1 ade2-1 can1-100 rdnaΔΔ::HIS3 carrying pNOY373-WT rRNA::LEU2*	(Leppek et al., 2020)
NOY890 tagged-hES9S	*MATA ura3-1 leu2-3,112 his3-11 trp1-1 ade2-1 can1-100 rdnaΔΔ::HIS3 carrying tagged pNOY373-rRNA-hES9S::LEU2*	(Leppek et al., 2020)
RPS2-FLAG	*MATA ura3-1 leu2-3,112 his3-11 trp1-1 ade2-1 can1-100 rdnaΔΔ::HIS3 RPS2-FLAG::kanMX6 carrying pRDN-hyg::URA3*	This study
RPS2-FLAG WT rRNA	*MATA ura3-1 leu2-3,112 his3-11 trp1-1 ade2-1 can1-100 rdnaΔΔ::HIS3 RPS2-FLAG::kanMX6 carrying pNOY373-WT rRNA-hES9S::LEU2*	This study
RPS2-FLAG tagged-hES9S	*MATA ura3-1 leu2-3,112 his3-11 trp1-1 ade2-1 can1-100 rdnaΔΔ::HIS3 RPS2-FLAG::kanMX6 carrying pNOY373-tagged rRNA-hES9S::LEU2*	This study

Table S3: DNA Oligonucleotides used in this study. Related to STAR Methods.

All DNA oligonucleotides used for cloning, RT-PCR, and qRT-PCR are listed in the table. F, forward primer; R, reverse primer.

Table S3. DNA oligonucleotides

Name	Sequence	Description
	qPCR primer	
KL050	TGGAGAATAACTTCTTCGTGGA	Rluc qPCR F
KL051	TTGGACGACGAACTTCACC	Rluc qPCR R
KL052	AAGAGATACGCCCTGGTTC	Fluc qPCR F
KL053	TTGTATTCAGCCCATATCGTTTC	Fluc qPCR R
KL318	TGCAAACTCCTTGGTCACAC	y-UsnRNA1(SNR19) qPCR F

KL319	CAAACTTCTCCAGGCAGAAG	y-UsnRNA1(SNR19) qPCR R
KL320	CCATCATGAAGTGTGATGTC	y-*actin1* qPCR F
KL321	GACCTTCATGGAAGATGGAG	y-*actin1* qPCR R
	qPCR primer for rRNA detection	
KL300	CTAGGCGAACAATGTTCTTAAAG	pre-mature 25S rRNA F
KL301	GACCTCAAATCAGGTAGGAGTACCC	mature 25S rRNA F
KL302	CACCGAAGGTACACTCGAGAGCTTC	tagged 25S rRNA R
KL303	CACCGAAGGTACCAGATTTC	endogenous 25S rRNA R
KL304	GCTTGTTGCTTCTTCTTTTAAGATAG	pre-mature 18S rRNA F
KL305	TACAGTGAAACTGCGAATGGC	mature 18S rRNA F
KL306	ATCTCTTCCAAAGGGTCGAG	endogenous 18S rRNA R
KL307	CGAGGATTCAGGCTTTGG	tagged 18S R
	PCR primer for rRNA strain characterization and ES9S sequencing	
KL314	GAACGAGACCTTAACCTACTAAATAGT	ES9S-span RT-PCR F
KL315	AAACCGATAGTCCCTCTAAGAAGT	ES9S-span RT-PCR R
KL316	GCTAATACATGCTTAAAATCTCGA	18Stag-span RT-PCR F
KL317	TTTTTATCTAATAAATACATCTCTTCCAA	18Stag-span RT-PCR R
KL473	TCGATTCCGTGGGTGGTGG	18S rRNA-seq primer F
KL474	TAGCGCGCGTGCAGC	18S rRNA-seq primer R
	In vitro transcription DNA template primer	
KL414	GCCGATTTAGGTGACACTATA**G**AAGAGctctggttgctctgtggg	IVT SP6-TIE primer F
KL415	CGGCATAAAGAATTGAAGAGAGTTTTCAC	IVT Fluc primer R
	5' UTR-specific PCR primer	
KL433	gagcaagggtgatctggccgGAATTCCTTTTCCTGTGGGAGCAGCC	T-*Rpl4* Gibson F
KL435	gagcaagggtgatctggccgGAATTCAAGAGGCTGGGGATTGCGTTA	T-*Hmgb2* Gibson F
KL437	gagcaagggtgatctggccgGAATTCAACACCCCTCCTAAGGCC	T-*Hba-x* Gibson F
KL438	gagcaagggtgatctggccgGAATTCTCGTCTCTATGGTTGCGCC	T-*Sem1* Gibson F
KL441	gagcaagggtgatctggccgGAATTCTCTCATTGTACAACCTTTCTTCAACTTCTTGT	T-*Id1* Gibson F
KL444	gagcaagggtgatctggccgGAATTCGGATTCTGCGTCCTCTCGC	T-*Cct5* Gibson F
KL445	gagcaagggtgatctggccgGAATTCTTCCTTTCTTAGCAGTTAACCGAGAGC	T-*Fubp1* Gibson F
KL447	gagcaagggtgatctggccgGAATTCGATGCCTGAGCATCACTCGC	T-*Pdcd5* Gibson F
KL449	ATGTTTTTGGCGTCTTCCATGACGGGGAGAGGAGAAGG	T-*Rpl4* Gibson R
KL451	ATGTTTTTGGCGTCTTCCATGACGACGGCGCGG	T-*Hmgb2* Gibson R
KL452	ATGTTTTTGGCGTCTTCCATTGGGAGGAGCGGCTC	T-*Rab10* Gibson R
KL453	ATGTTTTTGGCGTCTTCCATGGTGGTGGTGGTGGTGA	T-*Hba-x* Gibson R
KL454	ATGTTTTTGGCGTCTTCCATCGCGCCGCGCC	T-*Sem1* Gibson R
KL457	ATGTTTTTGGCGTCTTCCATGATCCTGAGAACAGGCGGAG	T-*Id1* Gibson R
KL460	ATGTTTTTGGCGTCTTCCATGGTGGACGAACTAGAACGAGC	T-*Cct5* Gibson R
KL461	ATGTTTTTGGCGTCTTCCATACCCACGCTACAGCACAC	T-*Fubp1* Gibson R
KL463	ATGTTTTTGGCGTCTTCCATGGCGCGGCTGTCC	T-*Pdcd5* Gibson R
KL466	CTCGAATCACTAGTCAGCTGGAATTC	pRF-EcoRI F Gibson
KL469	ATGTTTTTGGCGTCTTCCAT	Fluc-R Gibson
KL472	CTCGAATCACTAGTCAGCTGGAATTCGCACGGGCGCCG	EcoRI-*Rab10*(200nt) Gib. F
KL529	CTCGAATCACTAGTCAGCTGGAATTCAATGTTACAGAGCGGAGAGAGTGAG	*Hmgb1* Gib F
KL530	CTCGAATCACTAGTCAGCTGGAATTCGCTCTTTCCCCGCCCA	*Rpl18* Gib F
KL532	CTCGAATCACTAGTCAGCTGGAATTCGTCAGTGCGGCGGG	*Raly* Gib F
KL533	CTCGAATCACTAGTCAGCTGGAATTCGATTCCCTTCGGTCTTGCG	*Abcc5* Gib F
KL534	CTCGAATCACTAGTCAGCTGGAATTCGGCGGAGAGGCGG	*Maged1* Gib F
KL536	ATGTTTTTGGCGTCTTCCATGTTTAGTTGATTTTCCTCCGCGAGG	*Hmgb1* Gib R
KL537	ATGTTTTTGGCGTCTTCCATGATGGCGCCTCCTGCT	*Rpl18* Gib R

KL539	ATGTTTTTGGCGTCTTCCATGGTGTTCACCAGTACCAAGAATGAG	*Raly* Gib R
KL540	ATGTTTTTGGCGTCTTCCATCTTCACCACACAGAGGACCA	*Abcc5* Gib R
KL541	ATGTTTTTGGCGTCTTCCATAGCTCTCGTCTCCCTGG	*Maged1* Gib R
KL554	CTCGAATCACTAGTCAGCTGGAATTCCAGCCACTCTTTCTCACGTCG	*Rpl5* Gib F
KL555	CTCGAATCACTAGTCAGCTGGAATTCAGTTAAAAGGAGGTGCAGGGCC	*Gpx1* Gib F
KL556	CTCGAATCACTAGTCAGCTGGAATTCCTCAGCCCGTAGCCCG	*Tubb2b* Gib F
KL557	CTCGAATCACTAGTCAGCTGGAATTCAGTGCTGCTCCGGTACAGG	*Eno1* Gib F
KL558	ATGTTTTTGGCGTCTTCCATCCTGCGGAATAGAGACCCG	*Rpl5* Gib R
KL559	ATGTTTTTGGCGTCTTCCATCTCGGTGTAGTCCCGGATC	*Gpx1* Gib R
KL560	ATGTTTTTGGCGTCTTCCATGGTGCCTGGTTAGCTTCTTGC	*Tubb2b* Gib R
KL561	ATGTTTTTGGCGTCTTCCATGGCGAATTTCTGGCAGTAGGATC	*Eno1* Gib R
KL562	CTCGAATCACTAGTCAGCTGGAATTCGCTCTTTCCCCGCCCACTCCGGCGCGGTTCCGTC	*Rpl18*-full Gib F
KL563	GTTTTTGGCGTCTTCCATGATGGCGCCTCCTGCTCGGCCAGGTCCGGAAAGACGGAACCG	*Rpl18*-full Gib R
KL565	CTCGAATCACTAGTCAGCTGGAATTCTATAAAACCCGGCGGCGC	*ActB* Gib F
KL566	ATGTTTTTGGCGTCTTCCATGGCGAACTGGTGGCG	*ActB* Gib R
	Hybrid ES9S sequences	
24 nt	CCTACTAAATAGT**GGTGCTAGCATTTGCTGGTTATCC**ACTTCTTAGAGG	Yeast WT ES9S
31 nt	CCTACTAAATAGT**TACGCGACCCCCGAGCGGTCGGCGTCCCCCA**ACTTCTTAGAGG	hES9S
16 nt	AAAGCCTGAATCCTCG	18S rRNA sequence tag
24 nt	GGTACTGAAGCTCTCGAGTGTACC	25S rRNA sequence tag
